# Circulating tumor DNA as a biomarker of prognosis prediction in colorectal cancer: a systematic review and meta‐analysis

**DOI:** 10.1016/j.jncc.2024.05.007

**Published:** 2024-12-12

**Authors:** Qingxin Zhou, Xiaowei Chen, Baoqi Zeng, Meng Zhang, Nana Guo, Shanshan Wu, Hongmei Zeng, Feng Sun

**Affiliations:** 1 Department of Epidemiology and Biostatistics, School of Public Health, Peking University Health Science Centre, Beijing, China; 2 Tianjin Centers for Disease Control and Prevention, Tianjin, China; 3 Central Laboratory, Tianjin Fifth Central Hospital (Peking University Binhai Hospital), Tianjin, China; 4 Hebei Centers for Disease Control and Prevention, Hebei, China; 5 Clinical Epidemiology and EBM Unit, National Clinical Research Center for Digestive Diseases, Beijing Friendship Hospital, Capital Medical University, Beijing, China; 6 National Central Cancer Registry, National Cancer Center/National Clinical Research Center for Cancer/Cancer Hospital, Chinese Academy of Medical Sciences and Peking Union Medical College, Beijing, China; 7 Key Laboratory of Major Disease Epidemiology, Ministry of Education (Peking University), Beijing, China; 8 Xinjiang Medical University, Xinjiang Uygur Autonomous Region, China

**Keywords:** Colorectal cancer, Circulating tumor DNA, Prognostic biomarker, Prognosis prediction, Meta-analysis

## Abstract

**Objective:**

Circulating tumor DNA (ctDNA) is increasingly being used as a potential biomarker in colorectal cancer (CRC) patients. However, the role of ctDNA in CRC prognosis prediction remains unclear. The objective is to systematically assess the clinical value of ctDNA in colorectal cancer prognosis prediction throughout the treatment cycle.

**Methods:**

PubMed, Web of Science, Embase, Cochrane Library, Scopus, and clinical trials.gov database was searched from January 2016 to April 2023. Observational studies and randomized clinical trials reporting on ctDNA and prognostic outcomes in CRC patients were included. Pooled hazard risk ratios (HRs) were calculated for the primary outcomes, relapse-free survival (RFS), and overall survival (OS). Random-effects models were preferred considering the potential heterogeneity.

**Results:**

Sixty-five cohort studies were included. Association between ctDNA and shorter RFS or OS was significant, especially after the full-course treatment recommended by the guidelines (HR = 8.92 [ 95 % CI: 6.02–13.22], *P* < 0.001, *I^2^* = 73 %; HR = 3.05 [ 95 % CI: 1.72–5.41], *P* < 0.001, *I^2^* = 48 %) for all types of CRC patients. Despite the presence of heterogeneity, subgroup analyses showed that the cancer type and ctDNA detection assays may be the underlying cause. Besides, ctDNA may detect recurrence earlier than radiographic progression, but no uniform sampling time point between studies might bring bias. However, ctDNA detection did not appear to correlate with pathological complete response achievement in patients with locally advanced rectal cancer.

**Conclusion:**

ctDNA detection was significantly associated with poorer prognosis. The potential applications in prognostic prediction are promising and remain to be evaluated in other fields.

## 1 Introduction

Colorectal cancer (CRC) is the second leading cause of cancer-related deaths and ranks third in terms of incidence globally.^1^ More than 1.9 million new diagnosis and 0.9 million deaths were estimated to occur in 2020. The burden of CRC is projected to increase to 3.2 million in new cases and 1.6 million in deaths by 2040.^2^

There have been considerable advances in cancer treatment over the past few decades.^3^ Most early-stage CRCs are curable following surgery; however, approximately 5–30 % of patients face the risk of recurrence or progression in the postoperative period.^4^, ^5^, ^6^ For stage IV CRC, up to 65 % of patients may develop recurrent disease after systemic therapy.^5^^,^^7^ Studies have reported that the majority of the recurrences and metastases of CRCs occur within the first few years since the curative treatment, which suggested that minimal residual disease (MRD) or micro-metastases may exist.^8^, ^9^, ^10^ As recommended in the guidelines, patients need extensive follow-up to detect asymptomatic recurrences as early as possible to obtain optimal clinical treatment in the first 3 to 5 years.^11^, ^12^, ^13^ Therefore, identifying prognostic biomarkers for earlier disease progression is critical.

Commonly used follow-up biomarkers have shown varying degrees of limitations.^14^ For example, carcinoembryonic antigen (CEA) and computed tomography (CT) has a low sensitivity for CRC recurrence, and frequent radiation exposure could also pose a secondary risk to patients.^15^^,^^16^ To date, although the gold standard for clinical decision-making recommended by the guidelines^3^^,^^17^ is tumor biopsy, patients are generally reluctant to undergo colonoscopy due to the invasiveness and cumbersome preparation.^18^ On the basis of the above, liquid biopsy (LB) is gaining increasing attention as an alternative, non-invasive tool to circumvent these limitations.^19^ Circulating tumor DNA (ctDNA), which is shed into the blood by tumor and accounts for 0.01–90 % of the total circulating cell free DNA in the blood, is considered an important component.^20^ ctDNA can be detected through polymerase chain reaction (PCR) or next generation sequencing (NGS) assays via targeting tumor-specific mutations, structural variants, copy number alterations, and epigenetic features.^21^ At present, ctDNA based MRD detection has been well-established and widely used in haematological cancers, but remains challenging in solid tumors,^22^ although MRD detection has been mentioned in the National Comprehensive Cancer Network (NCCN) guidelines.^3^ Studies suggest that ctDNA detection presents opportunities for disease diagnosis and progression monitoring in CRC patients.^19^^,^^23^^,^^24^ However, previous reviews have primarily focused on the specific stages of CRC or single timepoint of ctDNA measurement, rather than its application throughout the entire treatment cycle for the broader CRC population.^25^, ^26^, ^27^ The role of ctDNA as a prognostic marker in CRC is currently no consensus on its optimal use in clinical practice.

Therefore, a comprehensive evaluation of the current literature is urgently needed. The aim of our study is to systematically summary the clinical value of ctDNA as a prognostic biomarker throughout the treatment cycle in different types of CRCs, potentially contributing to ctDNA's clinical utility.

## 2 Materials and methods

This study adhered to the Preferred Reporting Items for Systematic Reviews and Meta Analyses (PRISMA)^28^ and Meta-analysis of Observational Studies in Epidemiology (MOOSE) reporting guidelines (Supplementary Table 1).

### 2.1 Protocol and registration

The study protocol were prospectively registered on PROSPERO (CRD42022323474).

### 2.2 Search strategy

The electronic databases PubMed, Web of Science, Embase, Cochrane Library, Scopus, and clinicaltrials.gov were searched from January 2016 to May 2022, and ongoing clinical trials were tracked and included as soon as they were published until April 2023. Detailed search strategies are available in Supplementary Table 2. References of relevant articles were hand-searched.

### 2.3 Study selection

After removal of the duplicates, titles and abstracts were screened, and potentially eligible articles were reviewed based on the following eligibility and exclusion criteria. This process was performed independently by two authors and any discrepancies were resolved by discussion. Only conference abstracts published within the last three years were considered and studies with similar cohorts were carefully assessed to avoid repetition.

Studies were included if they met the following pre-specified criteria: (i) observational or randomized control studies; (ii) patients with colorectal cancer, both resectable and unresectable; (iii) documented collection and measurement of ctDNA (categorical variable, expressed as positive or negative). All timepoints and methods were allowed; (iv) prognostic outcomes such as relapse-free survival, overall survival, etc. reported; and (v) written in the English language.

Exclusion criteria included: (i) non-original studies or no primary data reported (e.g., reviews, editorials, comments, letters, or case reports); (ii) ctDNA was not divided into positive or negative categorical variables; (iii) other diseases; and (iv) diagnostic or screening outcomes.

### 2.4 Outcomes

The primary outcomes were relapse/disease/event/metastatic-free survival (abbreviated as RFS) and overall survival (OS). The RFS was defined as the time from inclusion or treatment to radiographic relapse, disease progression, or death. The OS was defined as the time from inclusion or treatment to death from any cause. Secondary outcomes included recurrence, pathological complete response (pCR), and lead time.

According to the guidelines,^3^^,^^11^^,^^29^ for early-stage CRC, the curative treatment mainly includes surgery. For resectable late-stage III or metastatic CRC (mCRC), postoperative adjuvant chemotherapy (ACT) is recommended as the standard of care. Preoperative neoadjuvant therapy (NAT) plus surgery is commonly used for those with locally advanced rectal cancer (LARC). Besides, systemic therapy is the most common treatment for unresectable late-stage CRC/mCRC. Based on these, the following ctDNA measurement timepoints were defined: baseline (before any treatment, all CRCs), during NAT (only for LARC), after NAT and before surgery (only for LARC), after surgery (only for resectable CRCs), after surgery and ACT (only for resectable CRCs and CRCs that required ACT), after full-course therapy recommended by the guidelines (after completing corresponding treatments, including surgery and chemotherapy for resectable CRCs, and systemic therapy for unresectable CRCs), and long-term post-treatment surveillance (all CRCs).

### 2.5 Data extraction

In this analysis, the following variables were extracted: (i) general information, including article title, author, year of publication, country of study, and sample size; (ii) population characteristics: age, sex, follow-up duration; (iii) disease characteristics: clinical stage and metastatic site; (iv) ctDNA measurements information: methods, timepoints, type of assay, definition of positivity; (v) outcome information: the effect values and their 95 % CIs for RFS and OS, the number of corresponding events in ctDNA positive and negative groups for recurrence and pCR, and the time lag of ctDNA test and radiographic examination for lead time.

### 2.6 Assessment of risk of bias

The risk of bias of randomized controlled trials was assessed by the Cochrane Collaboration's tool (RoB 2)^30^ and the Newcastle-Ottawa Scale (NOS) was used for observational studies.^31^ This process was performed independently by two authors and any discrepancies were resolved by discussion.

### 2.7 Data synthesis and statistical analysis

#### 2.7.1 Data synthesis and assessment of publication bias

The meta-analyses were conducted separately for each ctDNA measurement timepoint. A random-effects model was preferred considering the potential heterogeneity. Hazard ratios (HRs) with 95 % confidence intervals (CIs) were calculated for the RFS and OS outcome, the risk ratio (RR) for recurrence and odds ratio (OR) for pCR with 95 % CI was separately calculated. The significance of the pooled results was evaluated with a *Z*-test and the heterogeneity was assessed and reported using *I^2^* statistics (greater than 50 % considered as significant heterogeneity). All reported *P* values were two-sided, and *P* < 0.05 was considered statistically significant. Funnel plots and Egger's test were used to detect publication bias. All analyses were performed using R statistical software version 4.0.0 (R packages *metafor* and *meta*).

#### 2.7.2 Subgroup analyses and sensitivity analysis

Subgroup analyses were performed focusing on cancer types (CRC, LARC, and mCRC), assay types (tumor-inform versus tumor-agnostic) and resectability (resectable versus unresectable) for the primary outcomes. Difference between groups was tested using the Chi-square test. Sensitivity analyses were conducted using the leave-one-out method.

## 3 Results

### 3.1 Literature search and characteristics of included studies

**Fig. 1:**
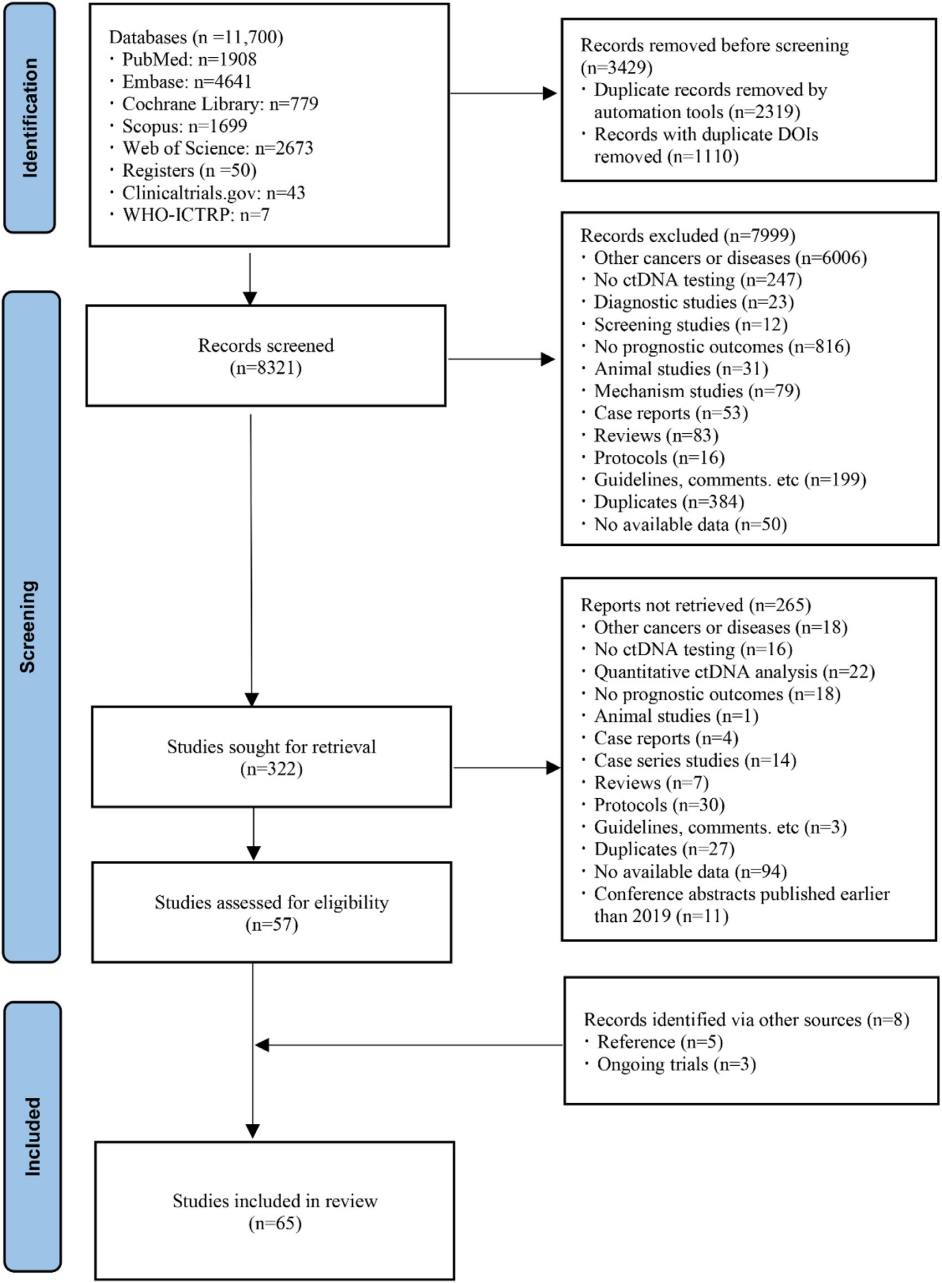
Flow diagram of studies inclusion and exclusion.

After screening and full-text reviewing, 65 articles were included, of which 11 were conference abstracts (Fig. 1). These studies incorporated CRC patients with or without metastases, of which 10 (15.38 %) were specific to LARC and four (6.15 %) to colon cancer. Common sites of metastasis included liver (*n* = 17; 26.15 %), lung (*n* = 3; 4.61 %), and peritoneum (*n* = 1; 1.54 %). Most studies were conducted in China (*n* = 11; 16.92 %), followed by Denmark (*n* = 9; 13.85 %), Australia (*n* = 7; 10.77 %), the United States (*n* = 7; 10.77 %), and Japan (*n* = 7; 10.77 %). Corresponding registration number or identification were mentioned in 23 studies. The sample sizes ranged from 23 to 1039, the proportion of male ranged from 46.00 % to 80.95 %, and the median age ranged from 54.0 to 71.3 years old. The median follow-up period ranged from 6.1 to 50.5 months (Table 1).

**Table 1:** Characteristics of the included studies.

Study ID	Country	Cancer Site	Trial ID	No. of Samples	Recruitment period	Age, mean±SD/median (IQR), years	Sex (male/female)	Clinical Stage	Follow-up, median (range)
Tie, 2016^32^	Australia	CC	ACTRN12612000326897	230	–	67 (32–80)	131/99	III	2 years
Ng, 2017^33^	Singapore	CRC	–	44	–		34/10	–	849 (91–1226) days
Schøler, 2017^34^	Denmark	mCRC-liver	–	45	–	–	30/15	I-IV	12.0 months
Murray, 2018^35^	Australia and New Zealand	CRC	ACTRN12611000318987	172	–	ctDNA^+^: 63 (95 % CI: 58–68)	105/67	III-IV	22.9 (IQR: 12.0–33.6) months
Benešová, 2019^36^	Czech	mCRC-liver	–	47	–	63.6 ± 12.3	31/16	–	–
Bidard, 2019^37^	France	mCRC-liver	NCT01442935, Prodige-14	153	Feb. 2011-Apr. 2015	60 (25–75)		–	37.2 (0–55.3) months
Huang, 2019^38^	China	CRC	–	43	Sept. 2016-May 2019	58 (30–77)	23/20	I-IV	120 (7–146) weeks
Tarazona, 2019^39^	Spain	CC	–	94	Oct. 2015-Oct. 2017	71.30±9.68	61/33	I-III	–
Tie, 2019^40^	Australia	CC	–	96	Nov. 2014-May 2017	71(48–93)	49/47	III	6.7–17.8 months
Tie, 2019^41^	Australia	LARC	ACTRN12612000327886	159	Apr. 2012-Dec. 2015	–	107/52	II-III	28.9 (11.6–46.4) months
Wang, 2019^42^	Swedish	CRC	–	58	Feb. 2007-May 2013	–	34/24	I-III	17.4 (6.6–28.7) months after surgery
Appelt, 2019^43^	Denmark	LARC	–	146	2005–2008	62 (33–75)	93/53	–	18.8 months
Elez, 2019^44^	Spain	mCRC	NCT01704703	29	–	59 (34–66)	19/10	–	5.1–10.6 years
Reinert, 2019^45^	Denmark	CRC	–	125	–	61 (29–75)	73/52	I-III	–
Wong, 2019^46^	Australia	mCRC	–	45	2013–2017	67 (48–85)	32/13	–	–
Beagan, 2020^47^	Netherlands	mCRC-peritonea	–	24	Aug. 2016-Mar.2018	65.2 ± 9.6	14/10	II-IV	–
Boysen, 2020^48^	Denmark	mCRC-liver/lung	–	35	Jul. 2015-Sept. 2017	70.5 (36–81)	18/17	–	21 months
Khakoo, 2020^49^	United Kingdom	LARC	NCT00825110	47	Feb. 2015-Nov. 2016	59 (30–83)	29/18	I-III	26.4 (IQR: 19.7–31.3) months
Murahashi, 2020^50^	Japan	LARC	–	85	Feb. 2017-Nov. 2018	60 (IQR: 52–69)	65/20	II-III	–
Suzuki, 2020^51^	Japan	CRC and GC	–	200	Mar. 2018-Mar. 2020	–	125/75	–	–
Tarazona, 2020*^,^^52^	–	CRC	–	193	–	ctDNA^+^: 63.4 ± 9.6	–	I-III	Over 2 years
Thomsen, 2020^53^	Denmark	mCRC	–	123	Mar. 2010-Nov. 2015	–	78/45	–	21.6 (4.6–38.5) months
Chan, 2020*^,^^54^	Japan	CRC	–	38	–	–	–	I-IV	–
Peng, 2020*^,^^55^	China	CRC	–	130	–	60 (35–84)	–	III	–
Anandappa, 2021*^,^^56^	United Kingdom	CRC	NCT04050345, TRACC	122	–			II-III	15.48 (0.16–42.1 months)
Benhaim, 2021^57^	France	CRC	NCT01198743, ALGECOLS	187	–	66.7 ± 11.3	108/79	II-III	–
Bolhuis, 2021^58^	Netherlands	mCRC-liver	NCT02162563, CAIRO5	23	Nov. 2014-Aug. 2018	63 (54–76)	15/8	–	19.6 (1.5–60) months
Chen, 2021^59^	China	CRC	–	240	Dec. 2017-Mar. 2020	60 (19–84)	136/104	II-III	27.4 (95 % CI 26.2–28.5) months
Ciardiello, 2021^60^	Italy	mCRC	NCT04561336, CAVE	77	Aug. 2018-Feb. 2020	–	42/35	–	–
Jin, 2021^61^	United States	CRC	–	82	Jan. 2016-June 2020	66 (33–85)	53/29	–	36–50 months
Knudsen, 2021^62^	Denmark	mCRC-liver	–	68	–	–	–	–	–
Kobayashi, 2021^63^	Japan	mCRC-liver	UMIN000034557	40	Jan. 2005-Dec. 2017	67 (40–87)	26/14	–	39 months
Lee, 2021^64^	Korea	CRC	–	49	Jan. 2018-Dec. 2019	64 (43–87)	23/26	–	31.4 (1.0–36.1) months
Loupakis, 2021^65^	Italy	mCRC-liver/lung	PREDATOR	112	–	60.1 (22.1–83.3)	72/40	–	10.7 (0.9–53.8) months
McDuff, 2021^66^	United States	LARC	–	29	Jan. 2014-Feb. 2018	54 (45–78)	15/14	II-III	20 (IQR: 14.0–43) months
Øgaard, 2021^67^	Denmark	mCRC-liver	–	96	May 2015-Dec. 2019	54 (IQR: 46–62)	64/32	–	6.1 (IQR: 3.1–9.6) months
Parikh, 2021^68^	United States	CRC	–	84	Aug. 2016-May 2019	67 (44–89)	51/33	I-IV	20.3 (IQR:13.8–34.9) months
Sefrioui, 2021^69^	France	mCRC-liver	–	192	Nov. 2010-Aug. 2014	ctDNA^+^: 66±9	108/84	IV	–
Taieb, 2021^70^	France	CC	–	1017	May 2009-May 2014	68 (27–87)	576/441	III	–
Unseld, 2021^71^	Switzerland	mCRC-liver/lung	–	30	Oct. 2017-Jul. 2018	ctDNA^+^: 63 (28–85)	20/10	–	–
Vidal, 2021^72^	Spain	LARC	GEMCAD 1402 Trial	62	Jan. 2015-Mar. 2017		40/22	–	41 (95 % CI: 32–50) months
Wang, 2021^73^	China	mCRC-liver	–	91	May 2018-Oct. 2019	65 (IQR: 59–73)	47/44	–	38 (2.3–51.5) months
Zhou, 2021^74^	China	LARC	NCT03042000	104	Aug. 2017-Feb. 2019	mean 60 (33–78)	67/37	II-III	49 (11–70) months
Wang, 2021^75^	China	LARC	NCT02605265	119	Feb. 2016-Oct. 2017	–	85/34	–	Minimum 36 months
Gu, 2021*^,^^76^	China	CRC	–	40	2017–2018	61 (29–75)	28/12	I-III	31 (6–42) months
Henriksen, 2021*^,^^77^	–	CRC	–	265	–	–	–	I-III	28.4 (1.2–51.0) months
Kotaka, 2021*^,^^78^	Japan	CRC	–	1365	Jun. 2020-Apr. 2021	–	–	I-IV	–
Hamfjord, 2021^79^	Norway	mCRC	NCT00145314, NORDIC-VII study	253	–	60 (26–74)	142/111	–	–
Ji, 2021^80^	China	LARC	–	46	Apr. 2014-Nov.2015	64 (57–69)	29/17	–	–
Tie, 2021^81^	Australia	mCRC-liver	ACTRN12612000345886	54	Jul. 2011-Dec. 2014	64.4 ± 12.5	38/16	–	–
Mason, 2021^82^	United States	mCRC-liver	–	63	Jan. 2016-Nov. 2018	57	32/31	–	50.5 (5–82) months
Symonds, 2021^83^	Australia	CRC	ACTRN12611000318987	69	–	69 (25–93)	46/23	I-III	12.5 (1.4–38.5) months
Henriksen, 2022^84^	Denmark/Spain	CRC	–	160	2014–2019	–	95/65	III	35 (IQR: 13–36) months
Liu, 2022^85^	China	LARC	NCT02533271, STELLAR	60	Dec. 2016-Oct. 2018	55 (29–68)	42/18	–	33.25 (9.63–42.43) months
Nishioka, 2022^86^	United States	mCRC-liver	–	105	Jan. 2013-Jun. 2020	45–77	63/42	–	28.3 (6.0−99.8) months
Tsukada, 2022*^,^^87^	Japan	CRC	COSMOS-CRC-01	93	–	64 (26–82)	–	II-III	27 (2–52) months
Li, 2022^88^	China	CRC	ChiCTR1800018754	165	Aug. 2018-Dec. 2019	62 (27–75)	–	–	–
Reinert, 2,022,^89^	Denmark	mCRC-liver	–	68	May. 2015–Dec. 2018	58.69±8.18	27/41	–	–
Grancher, 2022 ^90^	France	CRC	PRODIGE13 trial	134	2009–2013	64	69/42	II	16.74 (0.49–24.83) months
Chee, 2022*^,^^91^	United States	mCRC	–	45	–	–	–	–	50 (4–192) weeks
Lim, 2022*^,^^92^	–	mCRC	–	62	–	–	–	–	–
McNamara, 2022*^,^^93^	–	CRC	–	84	–	–	–	II-III	–
Gao, 2023^94^	China	mCRC-liver	–	124	Feb. 2017–Jul. 2020	61 (25–86)	81/43	–	30 (9–53) months
Kotani, 2023^95^	Japan	CRC	UMIN000039205, GALAXY	1039	Jun. 2020–Apr. 2021	67.7 (45.1–89.4)	550/489	II-III	36.7 (8.1–55.1) months
Reichert, 2,023^96^	United States	mCRC	–	223	Feb. 2018–Jun. 2021	55 (30–82)	126/97	–	6.5 years

⁎ conference abstract. Abbreviations: CC, colon cancer; CRC, colorectal cancer; GC, gastric cancer; LARC, localized advanced rectal cancer; mCRC, metastatic colorectal cancer; RC, rectal cancer; "-", not reported.

Different ctDNA measurement methods were also used. Twenty-two studies were PCR-based, 16 studies were NGS-based, 11 studies used both, and 1 used WES, while others did not report. In addition, tumor-inform assays were reported in 32 studies, 16 used tumor-agnostic assays, and 28 studies specified the definition of positivity for different ctDNA methods (Table 2 and Supplementary Table 3).

**Table 2:** Methodology and outcome information of the included studies.

Study ID	ctDNA detection method	Assay panel	Tumor-inform	Outcome	Lead time, median	NOS score
Tie, 2016^32^	PCR amplicon-based NGS	Safe-SeqS	Y	RFS\recurrence\lead time	167 (IQR: 81–279) days	9
Ng, 2017^33^	PCR	–	Y	Recurrence	–	7
Schøler, 2017^34^	ddPCR	–	Y	RFS\OS\recurrence\lead time	9.4 (95 % CI: 7.8–11) months	7
Murray, 2018^35^	qPCR	–	–	RFS\OS\recurrence	–	5
Benešová, 2019^36^	PCR	–	Y	Recurrence	–	7
Bidard, 2019^37^	ddPCR	–	N	OS	–	6
Huang, 2019^38^	–	EasyPure R Genomic DNA Kit	N	RFS\recurrence	–	5
Tarazona, 2019^39^	NGS	–	Y	RFS\recurrence\lead time	11.5 (range: 3–18) months	8
Tie, 2019^40^	PCR amplicon-based NGS	Safe-SeqS	Y	RFS\recurrence	–	9
Tie, 2019^41^	PCR amplicon-based NGS	Safe-SeqS	Y	RFS\recurrence\pCR	–	9
Wang, 2019^42^	PCR amplicon-based NGS	Safe-SeqS	Y	Recurrence\lead time	non-ACT: 4 (range: 2–31) months ACT: 1 (range: 1–1.25) month	8
Appelt, 2019^43^	–	–	–	RFS\pCR	–	7
Elez, 2019^44^	PCR	BEAMing	–	RFS\OS	–	5
Reinert, 2019^45^	PCR-NGS	–	Y	OS\lead time	8.7 (range: 0.8–16.5) months	9
Wong, 2019^46^	–	–	–	RFS	–	7
Beagan, 2020^47^	ddPCR	BioRad	Y	RFS\recurrence	–	7
Boysen, 2020^48^	ddPCR	UltraSEEK MA Colon Panel	N	RFS\recurrence	–	9
Khakoo, 2020^49^	ddPCR	–	–	RFS\OS\recurrence	–	6
Murahashi, 2020^50^	NGS	–	N	RFS\recurrence\pCR	–	5
Suzuki, 2020^51^	NGS/PCR	–	–	RFS	–	4
Tarazona, 2020^52^	PCR	–	Y	RFS\recurrence\lead time	9.08 (range: 0.56–16.5) months	4
Thomsen, 2020^53^	PCR	–	Y	RFS\OS	–	8
Chan, 2020^54^	NGS	Oncomine Pan-Cancer Panel	N	Lead time	3–3.5 months	3
Peng, 2020^55^	–	–	Y	RFS	–	5
Anandappa, 2021^56^	PCR amplicon-based NGS	Signatera™	Y	RFS\recurrence	–	5
Benhaim, 2021^57^	ddPCR	–	–	RFS\recurrence	–	7
Bolhuis, 2021^58^	ddPCR	–	N	RFS\recurrence\pCR	–	9
Chen, 2021^59^	NGS	Geneseeq Prime™ panel	Y	RFS\recurrence\lead time	5.01 months	9
Ciardiello, 2021^60^	qPCR	Idylla	–	RFS\OS	–	3
Jin, 2021^61^	qPCR	mqMSP	–	RFS\recurrence\lead time	8.0 (range: 0–12.5) months	5
Knudsen, 2021^62^	–	–	Y	Recurrence	–	3
Kobayashi, 2021^63^	–	Guardant360	N	RFS\recurrence	–	8
Lee, 2021^64^	–	–	–	RFS\recurrence	–	5
Loupakis, 2021^65^	PCR-NGS	–	Y	RFS\OS\recurrence\lead time	3.16 (range: 0.07–37.9) months	7
McDuff, 2021^66^	NGS	–	Y	Recurrence\pCR	–	6
Øgaard, 2021^67^	–	–	–	RFS\recurrence\lead time	3.1 (IQR: 2.7–7.1) months	7
Parikh, 2021^68^	–	Reveal™	N	RFS\recurrence	–	8
Sefrioui, 2021^69^	dPCR	–	Y	RFS\OS\recurrence	–	5
Taieb, 2021^70^	ddPCR	–	N	RFS\OS\recurrence	–	9
Unseld, 2021^71^	WGS	–	N	OS	–	6
Vidal, 2021^72^	NGS	–	–	RFS\OS\pCR	–	7
Wang, 2021^73^	NGS	HaploX Biotechnology		RFS\recurrence	–	8
Zhou, 2021^74^	NGS	–	Y	RFS\pCR	–	7
Wang, 2021^75^	NGS	–	Y	OS	–	5
Gu, 2021^76^	–	Super-Seq	N	RFS	–	5
Henriksen, 2021^77^	PCR and NGS		Y	RFS\lead time	8 (range: 0.56–21.6) months	4
Kotaka, 2021^78^	PCR amplicon-based NGS	Signatera™	Y	RFS	–	3
Hamfjord, 2021^79^	ddPCR	–	N	RFS\OS	–	7
Ji, 2021^80^	NGS	–	–	RFS\OS	–	4
Tie, 2021^81^	PCR amplicon-based NGS	Safe-SeqS	Y	RFS\recurrence	–	9
Mason, 2021^82^	–	Guardant360 CDx	Y	RFS\OS\recurrence	–	7
Symonds, 2021^83^	PCR	–	–	RFS		6
Henriksen, 2022^84^	Multiplex PCR based NGS		Y	RFS\recurrence\lead time	6 (IQR: 2–9) months	9
Liu, 2022^85^	–	–	Y	RFS\OS\recurrence\pCR\lead time	10.2 (range: 0.1–33.2) months	7
Nishioka, 2022^86^	NGS	Guardant Health	N	RFS\recurrence	–	7
Tsukada, 2022^87^	–	Reveal™	N	RFS\recurrence	–	6
Li, 2022^88^	NGS	–	Y	RFS\pCR	–	9
Reinert, 2022^89^	ddPCR	–	Y	RFS\recurrence\lead time	2.5 (95 % CI: 1.2–3.9) months	8
Grancher, 2022^90^	ddPCR	BioRad	Y	RFS\recurrence	–	9
Chee, 2022^91^	–	Reveal™	N	RFS\recurrence\lead time	28 weeks	5
Lim, 2022^92^	NGS	–	N	Lead time	3.3 months	3
McNamara, 2022^93^	–	–	–	Recurrence	–	4
Gao, 2023^94^	NGS	–	Y	OS	–	8
Kotani, 2023^95^	NGS	Signatera™	Y	RFS\recurrence	–	9
Reichert, 2023^96^	NGS	Foundation One	–	OS\recurrence	–	5

Abbreviations: ddPCR, droplet digital PCR; IQR, interquartile range; NGS, next-generation sequencing; N, no; NOS, Newcastle-Ottawa scale; OS, overall survival; pCR, pathological complete response; PCR, polymerase chain reaction; RFS, recurrence-free survival; WES, whole genome re-sequencing; Y, yes; "-", not reported.

### 3.2 Risk of bias

Among cohort studies included, the overall risk of bias score ranged from 3 to 9; 36 studies were classified as low-risk (scores: 7–9), 24 studies were moderate-risk (scores: 4–6), and the reminders were high-risk (scores: 3). The majority of high-risk studies were limited by insufficient information (Supplementary Table 4).

### 3.3 ctDNA and RFS

**Fig. 2:**
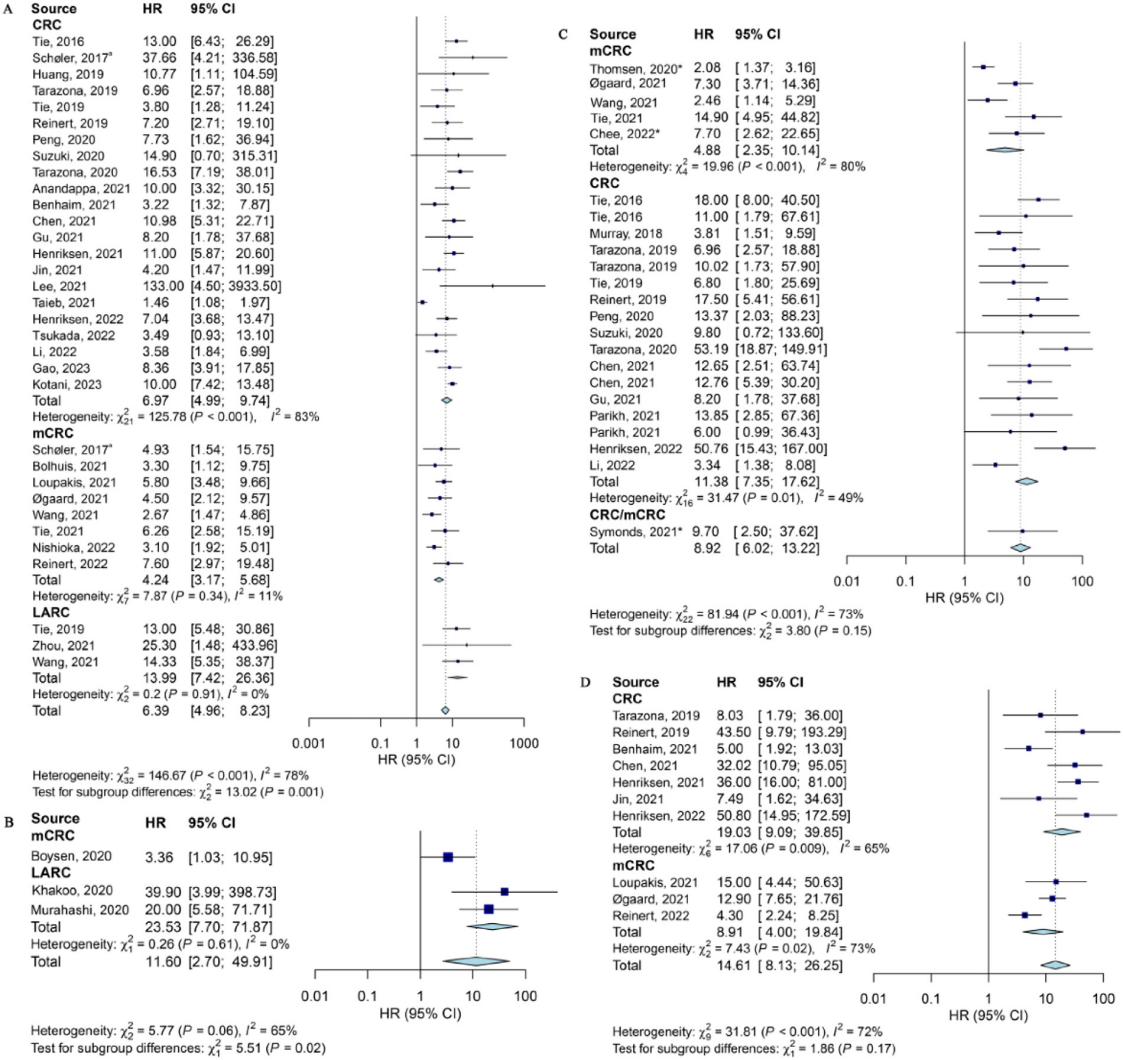
Forest plot of the association between ctDNA detection at different timepoint with RFS. (A) Just after surgery (only for resectable CRCs); (B) After surgery and ACT (only for resectable and CRCs required ACT); (C) After full-course therapy (after completing corresponding treatments, all CRCs); and (D) after long-term post-treatment surveillance (all CRCs). *, unresectable CRC. ACT, adjuvant chemotherapy; CRC, colorectal cancer (early-stage); HR, hazard ratio; LARC, locally advanced rectal cancer; mCRC, metastatic CRC; RFS, relapse-free survival.

ctDNA measured at different timepoints were significantly associated with worse RFS (Fig. 2 and Supplementary Fig. 1). For resectable CRCs, 33 studies reported the association between ctDNA measured just after surgery (HR = 6.39, 95 % CI: 4.96–8.23, *P* < 0.001; *I^2^*=78 %, Fig. 2A). Further, for those need ACT after surgery, results were still significant in both mCRC and LARC groups (Fig. 2B). For both resectable and unresectable CRCs, after the full-course treatment, HR was 8.92 (95 % CI: 6.02–13.22, *P* < 0.001; *I^2^* = 73 %, Fig. 2C) for 23 studies. The significance risk of worse RFS persisted through the long-term post-treatment surveillance period (Fig. 2D)**.**

### 3.4 ctDNA and OS

**Fig. 3:**
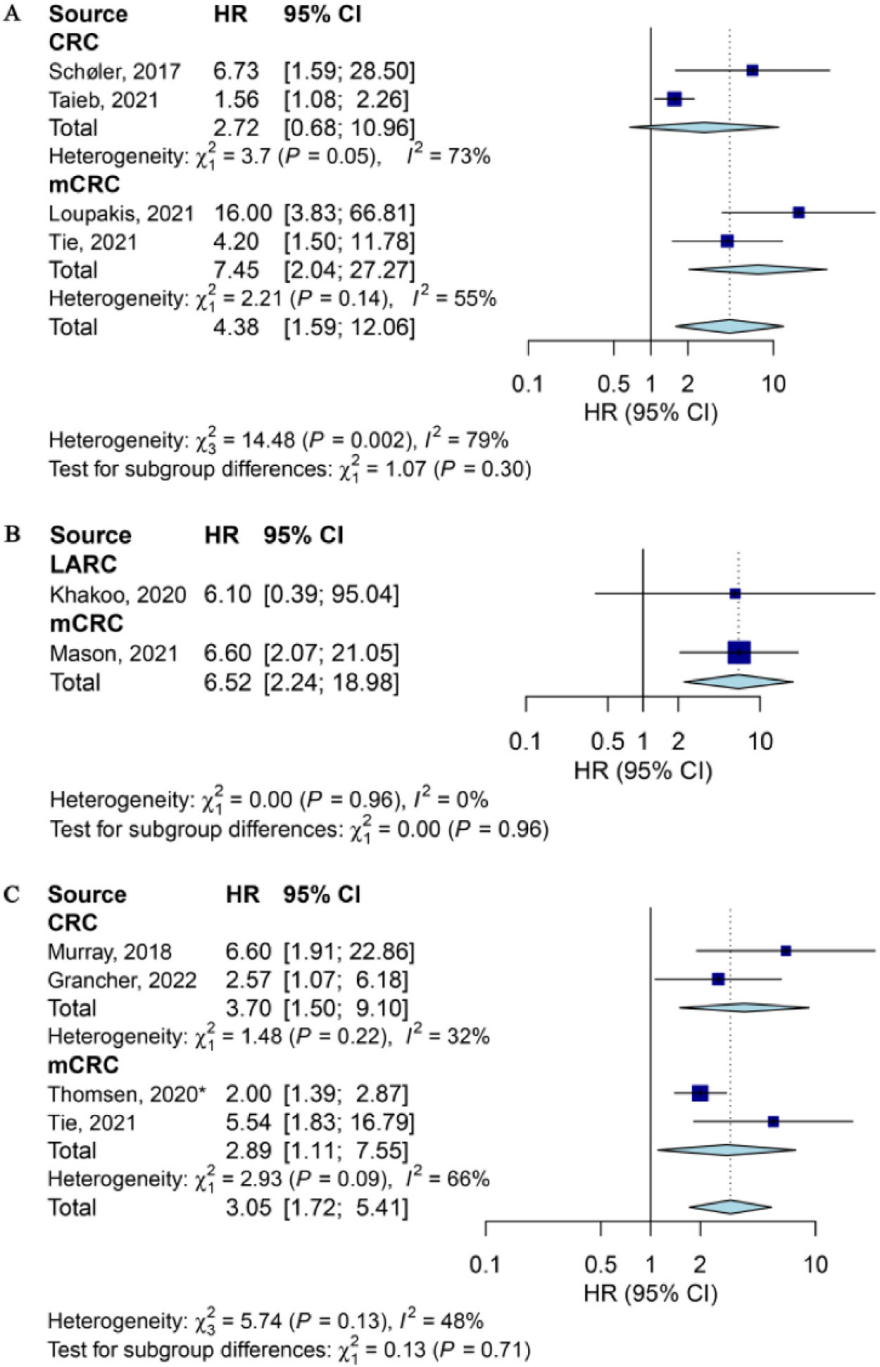
Forest plot of the association between ctDNA detection at different timepoints with OS. (A) Just after surgery (only for resectable CRCs); (B) After surgery and ACT (only for resectable and CRCs required ACT); and (C) After full-course therapy (after completing corresponding treatments, all CRCs). *, unresectable colorectal cancer. ACT, adjuvant chemotherapy; CRC, colorectal cancer (early stage); CI, confidence interval; HR, hazard ratio; LARC, locally advanced rectal cancer; mCRC, metastatic CRC; OS, overall survival.

Association between poor OS and ctDNA detection persisted throughout the whole treatment cycle (Fig. 3 and Supplementary Fig. 2). In resectable CRC patients, HR was 4.38 (95 % CI: 1.59–12.06, *P* = 0.004; *I^2^*= 79 %, Fig. 3A) after surgery and 6.52 (95 % CI: 2.24–18.98, *P* < 0.001; *I^2^* = 0 %, Fig. 3B) for those who received ACT subsequently. Four studies reported the significant association between ctDNA measured after the full-course treatment and OS (Fig. 3C).

### 3.5 ctDNA and recurrence

Studies reported that patients with positive ctDNA at any detection time had a high risk of recurrence (Supplementary Fig. 3). RRs were found to be increased from post-surgery (RR = 3.15, 95 % CI: 2.56–3.87, *P* < 0.001; *I^2^* = 83 %), post-full-course treatment (RR = 4.32, 95 % CI: 2.95–6.31, *P* < 0.001; *I^2^* = 75 %) to long-term post-treatment surveillance (RR = 6.05, 95 % CI: 2.65–13.83, *P* < 0.001; *I^2^*= 84 %).

### 3.6 Lead time

**Fig. 4:**
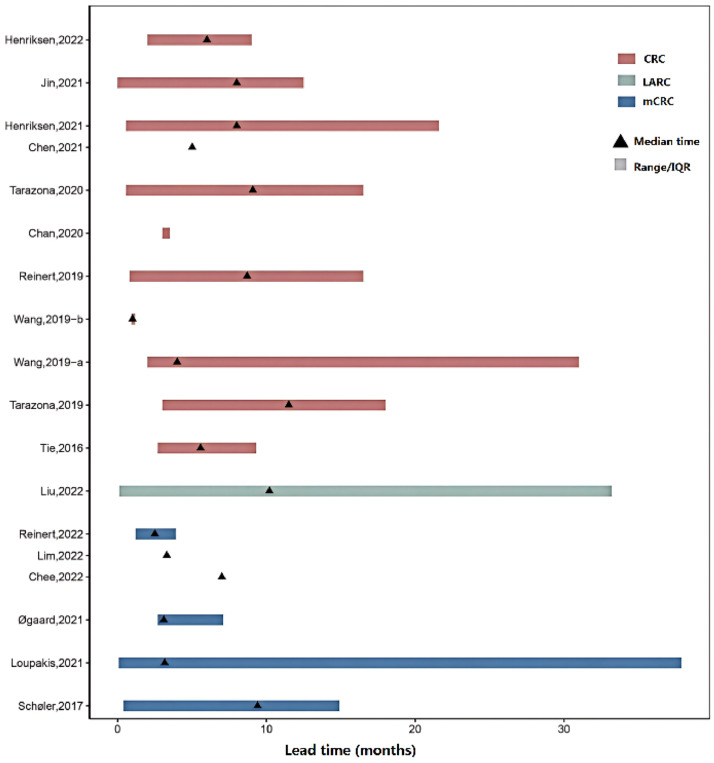
Plot of ctDNA lead time compared with the imaging recurrence in different types of CRCs. CRC, early-stage colorectal cancer; IQR, interquartile range; LARC, locally advanced rectal cancer; mCRC, metastatic CRC. (For interpretation of the references to colour in this figure legend, the reader is referred to the web version of this article.)

Seventeen studies reported lead time, generally defined as the time from ctDNA detection to radiographic recurrence, with the median time ranging from 1.0 to 11.5 months. In addition, lead time varied across different cancer types, with median time of 1.0–11.5 months, 10.2 months, and 3.1–9.4 months for CRC, LARC, and mCRC, respectively, (Fig. 4). Difference also existed between different assay types, with median time of 1.0–11.5 months and 3.0–8.0 months for tumor-inform and tumor-agnostic assays, respectively. Besides, only seven studies reported the frequency of ctDNA sampling or radiographic imaging during follow-up, with ctDNA sampling every 3.0–6.0 months on average and imaging every 6.0–12.0 months on average.

### 3.7 ctDNA and pCR

Six studies of LARC reported that no significant association was found between the presence of ctDNA and pCR achievement, either at baseline or before surgery (Supplementary Fig. 4).

### 3.8 Subgroup analysis

Subgroup analyses revealed that a greater association was found between ctDNA measured after surgery and RFS in the LARC group (HR = 13.99, 95 % CI: 7.42–26.36, *I^2^* = 0 %, Fig. 2**A**) with no heterogeneity. After grouping according to resectability or not, heterogeneity intra-group was reduced and the resected group showed worse RFS risk (HR = 10.04, 95 % CI: 6.69–15.06, *I^2^* = 58 %, Supplementary Table 5). Subgroup analyses stratified by assay type eliminated intra-group heterogeneity and reported stronger association between ctDNA detected after surgery and poor OS in the tumor-informed group (HR = 6.82, 95 % CI: 3.10–15.01, *I^2^* = 10 %, Supplementary Table 5).

### 3.9 Sensitivity analysis and publication bias

The results of RFS and OS between different timepoints were robust and no significant changes were found in the sensitivity analysis (Supplementary Figs. 5 and 6). Sensitivity analysis of recurrence and pCR were shown in Supplementary Figs. 7 and 8. Visual inspection of the funnel plots and the Eggers’ test results suggested that there was publication bias in RFS when ctDNA was detected after surgery or after full-course treatment (*P* < 0.05, Supplementary Fig. 9). The Duval trim and fill procedure was conducted to assess publication bias. The pooled HRs for the two results were 3.57 (95 % CI: 2.60–4.92, *P* < 0.001) and 4.22 (95 % CI: 2.63–6.80, *P* < 0.001) after the missing studies were imputed, indicating a significant association still existed.

## 4 Discussion

In this systematic review, 65 articles were eligible for meta-analysis and showed that ctDNA detection during the cycle of CRC treatment is associated with poorer prognosis, especially poorer RFS and OS. To the best of our knowledge, this review presents a comprehensive effort to encompass all disease stages, full-cycle ctDNA detection, and multiple outcomes simultaneously.

### 4.1 Association of ctDNA and prognostic outcome

Our findings concur with previously published studies. One study^97^ reported that high baseline ctDNA level was associated with short RFS and OS in metastatic CRC. Callesen and colleagues^25^ reported that ctDNA detected across the NAT or the ACT period both was associated with poor survival outcomes. The study by Dizdarevic et al.^98^ showed a negative correlation between ctDNA positivity at baseline, before and after surgery and prognosis. In addition, three studies^27^^,^^99^^,^^100^ that included different stages of CRC patients demonstrated that positive ctDNA after surgery had a significant value in prognosis. Besides, strong associations between ctDNA and recurrence were also shown in unresectable CRC patients after full-course treatment. As even a few remaining cancer cells can continue to multiply and eventually cause a recurrence, ctDNA tests were significantly correlated with prognosis by quantifying the small number of cancer cells.

No significant association between ctDNA and pCR was found in our study, but Marina and colleagues^23^ suggested that undetectable baseline ctDNA might be predictive of achieving pCR in LARC. Given that no quantitative analysis was provided, more research is needed to explore the true association.

### 4.2 Lead time between ctDNA and radiographic imaging

Seventeen studies indicated that ctDNA may precede radiographic progression in detecting recurrence, for it represented the molecular progression, which is also consistent with other reviews.^16^^,^^97^ However, it should be noted that the lead time was influenced by the frequency of follow-up and sample collection, which could vary considerably between studies, so it remains to be seen whether ctDNA works better than radiological assessment.

### 4.3 Heterogeneity among different studies

There is some heterogeneity existed in our results. After the subgroup analyses, some heterogeneity was decreased or eliminated, but some remained, especially in the CRC group. Among those studies, differences in study designs may account for the greatest cause, due to the varied treatment and follow-up time. Besides, we used the compound outcomes in RFS analyses, which may also introduce confounders. In addition, although we performed subgroup analyses according to the ctDNA detection assay, different measurement strategies with different testing thresholds may also affect the results, given that standardized methods are lacking.

### 4.4 Strengths and limitations

There are some strengths in this review. First, we examined the association of ctDNA with prognostic outcomes at multiple timepoints of treatment. Second, different subgroup analyses were performed to explore heterogeneity, and effects were compared between groups. Thirdly, sensitivity analyses were conducted to confirm the stability of results.

Our study still has some limitations. First, we reviewed literature from 2016 to 2023. Taking into account the fact that the first liquid biopsy test was approved by the FDA in 2016,^101^ this relatively short study period may result in an inexhaustive review. However, another review^26^ indicated that the majority of studies were published after 2018, which aligns with our design and minimizes this potential impact. Second, only studies that reported qualitative testing of ctDNA were included. It remains unclear to what extent changes in quantitative ctDNA levels impact the prognosis results. Third, publication bias existed especially in the analysis of association between ctDNA and RFS outcome. Although publication bias may have affected the observed results, the results remain stable after using the trim method.

### 4.5 Further research

Several studies^75^, ^88^, ^94^ have attempted to establish prognostic risk prediction models using ctDNA in conjunction with other clinical factors to improve prediction accuracy, and research^102^^,^^103^ into the value of ctDNA in precision therapy is still being explored. In addition, the cost-effectiveness of ctDNA detection needs to be assessed in terms of patient benefit and societal healthcare costs.

## 5 Conclusion

In conclusion, this study found that ctDNA detection was significantly associated with poorer prognosis in CRC patients, especially after full-course treatment. The potential applications of ctDNA in prognostic prediction are promising. Further research and technological developments are still needed to evaluate the cost-effectiveness and clinical utility of ctDNA in guiding treatment.

## Declaration of competing interests

The authors declare that they have no known competing financial interests or personal relationships that could have appeared to influence the work reported in this paper.

## Appendix Supplementary materials

Image, application 1
