# Supplementary material for: Circulating tumor DNA as a biomarker of prognosis prediction in colorectal cancer: a systematic review and meta‐analysis
Source: J Natl Cancer Cent. 2024 Dec 12;5(2):167–78. doi: 10.1016/j.jncc.2024.05.007 (PMC12010414; doi:10.1016/j.jncc.2024.05.007)
Supplement: Supplementary file 1 [file mmc1.pdf]

Supplementary Materials

(Unedited, provided by the authors.)

Circulating Tumor DNA (ctDNA) as A Biomarker of Prognosis Prediction in Colorectal Cancer: A Systematic Review and Meta-analysis

Qingxin Zhou; Xiaowei Chen; Baoqi Zeng; Meng Zhang; Nana Guo; shanshan Wu; Hongmei Zeng; Feng Sun

Supplementary Materials 1
Supplementary Table 1 PRISMA 2020 Checklist and MOOSE Checklist for Meta-analyses of Observational Studies 1
Supplementary Table 2 Search strategy and results 5
Supplementary Table 3 Exposure and outcome measurement 8
Supplementary Table 4 Risk of Bias 20
Supplementary Table 5 Subgroup analysis 24
Supplementary Fig. 1 Forest plot of the association between ctDNA detection and RFS before treatment. 29
Supplementary Fig. 2 Forest plot of the association between ctDNA detection and OS before treatment. 30
Supplementary Fig. 3. Forest plot of the association between ctDNA detection and recurrence. 31
Supplementary Fig. 4. Forest plot of the association between ctDNA detection and pCR. 32
Supplementary Fig. 5 Sensitivity analysis of the association between ctDNA detection and RFS using leave-one-out methods. 33
Supplementary Fig. 6 Sensitivity analysis of the association between ctDNA detection and OS using leave-one-out methods. 34
Supplementary Fig. 7 Sensitivity analysis of the association between ctDNA detection and recurrence using leave-one-out methods. 35
Supplementary Fig. 8 Sensitivity analysis of the association between ctDNA detection and pCR using leave-one-out methods. 36
Supplementary Fig. 9 Funnel plot of the association between ctDNA detection at and RFS. 37
Supplementary Fig. 10 Funnel plot of the association between ctDNA detection at baseline (before any treatment, all CRCs) and OS. 38
Supplementary Fig. 11 Funnel plot of the association between ctDNA detection and recurrence. 39

Supplementary Table 1 PRISMA 2020 Checklist and MOOSE Checklist for Meta-analyses of Observational Studies

Table 1.1 PRISMA 2020 checklist

Section and Topic	Item #	Checklist item	Location where item is reported
**TITLE**			
Title	1	Identify the report as a systematic review.	1
**ABSTRACT**			
Abstract	2	See the PRISMA 2020 for Abstracts checklist.	2
**INTRODUCTION**			
Rationale	3	Describe the rationale for the review in the context of existing knowledge.	5-6
Objectives	4	Provide an explicit statement of the objective(s) or question(s) the review addresses.	7
**METHODS**			
Eligibility criteria	5	Specify the inclusion and exclusion criteria for the review and how studies were grouped for the syntheses.	6-7
Information sources	6	Specify all databases, registers, websites, organisations, reference lists and other sources searched or consulted to identify studies. Specify the date when each source was last searched or consulted.	6
Search strategy	7	Present the full search strategies for all databases, registers and websites, including any filters and limits used.	6
Selection process	8	Specify the methods used to decide whether a study met the inclusion criteria of the review, including how many reviewers screened each record and each report retrieved, whether they worked independently, and if applicable, details of automation tools used in the process.	6
Data collection process	9	Specify the methods used to collect data from reports, including how many reviewers collected data from each report, whether they worked independently, any processes for obtaining or confirming data from study investigators, and if applicable, details of automation tools used in the process.	7-8
Data items	10a	List and define all outcomes for which data were sought. Specify whether all results that were compatible with each outcome domain in each study were sought (e.g. for all measures, time points, analyses), and if not, the methods used to decide which results to collect.	7
	10b	List and define all other variables for which data were sought (e.g. participant and intervention characteristics, funding sources). Describe any assumptions made about any missing or unclear information.	7-8
Study risk of bias assessment	11	Specify the methods used to assess risk of bias in the included studies, including details of the tool(s) used, how many reviewers assessed each study and whether they worked independently, and if applicable, details of automation tools used in the process.	8
Effect measures	12	Specify for each outcome the effect measure(s) (e.g. risk ratio, mean difference) used in the synthesis or presentation of results.	8
Synthesis methods	13a	Describe the processes used to decide which studies were eligible for each synthesis (e.g. tabulating the study intervention characteristics and comparing against the planned groups for each synthesis (item #5)).	8
	13b	Describe any methods required to prepare the data for presentation or synthesis, such as handling of missing summary statistics, or data conversions.	8
	13c	Describe any methods used to tabulate or visually display results of individual studies and syntheses.	8
	13d	Describe any methods used to synthesize results and provide a rationale for the choice(s). If meta-analysis was performed, describe the model(s), method(s) to identify the presence and extent of statistical heterogeneity, and software package(s) used.	8
	13e	Describe any methods used to explore possible causes of heterogeneity among study results (e.g. subgroup analysis, meta-	8

Section and Topic	Item #	Checklist item	Location where item is reported
		regression).	
Synthesis methods	13f	Describe any sensitivity analyses conducted to assess robustness of the synthesized results.	9
Reporting bias assessment	14	Describe any methods used to assess risk of bias due to missing results in a synthesis (arising from reporting biases).	8
Certainty assessment	15	Describe any methods used to assess certainty (or confidence) in the body of evidence for an outcome.	-
**RESULTS**			
Study selection	16a	Describe the results of the search and selection process, from the number of records identified in the search to the number of studies included in the review, ideally using a flow diagram.	9
	16b	Cite studies that might appear to meet the inclusion criteria, but which were excluded, and explain why they were excluded.	
Study characteristics	17	Cite each included study and present its characteristics.	9
Risk of bias in studies	18	Present assessments of risk of bias for each included study.	9
Results of individual studies	19	For all outcomes, present, for each study: (a) summary statistics for each group (where appropriate) and (b) an effect estimate and its precision (e.g. confidence/credible interval), ideally using structured tables or plots.	10-11
Results of syntheses	20a	For each synthesis, briefly summarise the characteristics and risk of bias among contributing studies.	10-11
	20b	Present results of all statistical syntheses conducted. If meta-analysis was done, present for each the summary estimate and its precision (e.g. confidence/credible interval) and measures of statistical heterogeneity. If comparing groups, describe the direction of the effect.	10-11
	20c	Present results of all investigations of possible causes of heterogeneity among study results.	10-11
	20d	Present results of all sensitivity analyses conducted to assess the robustness of the synthesized results.	11
Reporting biases	21	Present assessments of risk of bias due to missing results (arising from reporting biases) for each synthesis assessed.	-
Certainty of evidence	22	Present assessments of certainty (or confidence) in the body of evidence for each outcome assessed.	-
**DISCUSSION**			
Discussion	23a	Provide a general interpretation of the results in the context of other evidence.	11-14
	23b	Discuss any limitations of the evidence included in the review.	13
	23c	Discuss any limitations of the review processes used.	13
	23d	Discuss implications of the results for practice, policy, and future research.	14
**OTHER INFORMATION**			
Registration and protocol	24a	Provide registration information for the review, including register name and registration number, or state that the review was not registered.	6
	24b	Indicate where the review protocol can be accessed, or state that a protocol was not prepared.	6
	24c	Describe and explain any amendments to information provided at registration or in the protocol.	-
Support	25	Describe sources of financial or non-financial support for the review, and the role of the funders or sponsors in the review.	-
Competing interests	26	Declare any competing interests of review authors.	-
Availability of data, code and other materials	27	Report which of the following are publicly available and where they can be found: template data collection forms; data extracted from included studies; data used for all analyses; analytic code; any other materials used in the review.	-

Table 1.2 MOOSE checklist for meta-analyses of observational studies

Item No	Recommendation	Reported on Page No
**Reporting of background should include**		
1	Problem definition	5
2	Hypothesis statement	7
3	Description of study outcome(s)	7
4	Type of exposure or intervention used	6-7
5	Type of study designs used	6
6	Study population	6
**Reporting of search strategy should include**		
7	Qualifications of searchers (eg, librarians and investigators)	6
8	Search strategy, including time period included in the synthesis and keywords	6
9	Effort to include all available studies, including contact with authors	6
10	Databases and registries searched	6
11	Search software used, name and version, including special features used (eg, explosion)	6
12	Use of hand searching (eg, reference lists of obtained articles)	6
13	List of citations located and those excluded, including justification	-
14	Method of addressing articles published in languages other than English	6
15	Method of handling abstracts and unpublished studies	6
16	Description of any contact with authors	-
**Reporting of methods should include**		
17	Description of relevance or appropriateness of studies assembled for assessing the hypothesis to be tested	6-7
18	Rationale for the selection and coding of data (eg, sound clinical principles or convenience)	6-7
19	Documentation of how data were classified and coded (eg, multiple raters, blinding and interrater reliability)	6-7
20	Assessment of confounding (eg, comparability of cases and controls in studies where appropriate)	-
21	Assessment of study quality, including blinding of quality assessors, stratification or regression on possible predictors of study results	8
22	Assessment of heterogeneity	8
23	Description of statistical methods (eg, complete description of fixed or random effects models, justification of whether the chosen models account for predictors of study results, dose-response models, or cumulative meta-analysis) in sufficient detail to be replicated	8
24	Provision of appropriate tables and graphics	8
**Reporting of results should include**		
25	Graphic summarizing individual study estimates and overall estimate	9-11
26	Table giving descriptive information for each study included	9
27	Results of sensitivity testing (eg, subgroup analysis)	11
28	Indication of statistical uncertainty of findings	9-11
**Reporting of discussion should include**		
29	Quantitative assessment of bias (eg, publication bias)	11

Item No	Recommendation	Reported on Page No
30	Justification for exclusion (eg, exclusion of non-English language citations)	-
31	Assessment of quality of included studies	9
**Reporting of conclusions should include**		
32	Consideration of alternative explanations for observed results	11-13
33	Generalization of the conclusions (ie, appropriate for the data presented and within the domain of the literature review)	11-13
34	Guidelines for future research	14
35	Disclosure of funding source	-

From: Stroup DF, Berlin JA, Morton SC, et al, for the Meta-analysis Of Observational Studies in Epidemiology (MOOSE) Group. Meta-analysis of Observational Studies in Epidemiology. A Proposal for Reporting. *JAMA*. 2000;283(15):2008-2012. doi: 10.1001/jama.283.15.2008..

Supplementary Table 2 Search strategy and results

Number of citations by each database and trial register searched (2016.1-2022.5)

Databases and trial registers	Citations
**Databases**	
PubMed	1908
Embase	4641
Cochrane Library	779
Clinicaltrial.gov	43
WHO-ICTRP	7
The Web of science	2673
Scopus	1699
**Total**	**11750**

1. Search strategy for Pubmed

((((("neoplasms"[MeSH Terms] OR "breast cancer"[Title/Abstract] OR "lung cancer"[Title/Abstract] OR "colorectal cancer"[Title/Abstract] OR "colon cancer"[Title/Abstract] OR "rectal cancer"[Title/Abstract] OR "esophageal cancer"[Title/Abstract] OR "gastric cancer"[Title/Abstract] OR "liver cancer"[Title/Abstract] OR "hepatocellular carcinoma"[Title/Abstract] OR "pancreatic cancer"[Title/Abstract] OR "cholangiocarcinoma"[Title/Abstract] OR "gallbladder carcinoma"[Title/Abstract]) NOT ("hematologic neoplasms"[MeSH Major Topic] OR "neoplasms, plasma cell"[MeSH Major Topic] OR "multiple myeloma"[MeSH Major Topic] OR "leukemia"[MeSH Major Topic] OR "lymphoma"[MeSH Major Topic] OR "myelodysplastic syndromes"[MeSH Major Topic] OR "leukemia"[Title/Abstract] OR "myeloma"[Title/Abstract] OR "lymphoma"[Title/Abstract] OR "lymphoproliferative"[Title/Abstract] OR "myeloid"[Title/Abstract] OR "lymphoma*"[Title] OR "Hodgkin"[Title] OR "Immunoproliferative Small Intestinal"[Title/Abstract] OR "AML"[Title] OR "CLL"[Title] OR "CML"[Title] OR "APL"[Title] OR "HL"[Title] OR "NHL"[Title] OR "hematopoietic*"[Title] OR "haematologic*"[Title])) AND (("Circulating Tumor DNA"[MeSH Terms] OR "Circulating Tumor DNA"[Title/Abstract] OR "ctDNA"[Title/Abstract] OR "ct DNA"[Title/Abstract] OR ("molecular residual disease*"[Title/Abstract] OR "neoplasm, residual"[MeSH Major Topic] OR "minimal residual"[Title/Abstract] OR "MRD"[Title/Abstract] OR "residual minimal"[Title/Abstract] OR "measurable residual"[Title/Abstract])) NOT ("Chlamydia trachomatis"[MeSH Terms] OR "Chlamydia trachomatis"[Text Word] OR "calf thymus dna"[Supplementary Concept] OR "calf thymus"[Text Word])) AND ("Observational Study"[Publication Type] OR "Observational Studies as Topic"[MeSH Terms] OR "Cohort Studies"[MeSH Terms] OR "Case-Control Studies"[MeSH Terms] OR "Cross-Sectional Studies"[MeSH Terms] OR "observational stud*"[Title/Abstract] OR "Cohort"[Title/Abstract] OR "Follow-Up"[Title/Abstract] OR "longitudinal*"[Title/Abstract] OR "prospectiv*"[Title/Abstract] OR "retrospectiv*"[Title/Abstract] OR "Case-Control"[Title/Abstract] OR "Cross-Sectional"[Title/Abstract] OR "case series"[Title/Abstract] OR "single arm"[Title/Abstract] OR ("controlled clinical trial"[Publication Type] OR "Controlled Clinical Trials as Topic"[MeSH Terms] OR "Random Allocation"[MeSH Terms] OR "Double-Blind Method"[MeSH Terms] OR "single-blind method"[MeSH Terms] OR "Control Groups"[MeSH Terms] OR "cross-over studies"[MeSH Terms] OR "random*"[Title/Abstract] OR "placebo"[Title/Abstract] OR "trial"[Title/Abstract] OR "groups"[Title/Abstract] OR "crossover"[Title/Abstract] OR "cross-over"[Title/Abstract]))) NOT ("Animals"[MeSH Terms] NOT ("Humans"[MeSH Terms] AND "Animals"[MeSH Terms]))) NOT ("Review"[Title] OR "Review"[Title] OR "Meta"[Title] OR "Meta"[Title])) AND (2016:2022[pdat])

2. Search strategy for Embase

((('neoplasm'/exp OR 'breast cancer':ti,ab OR 'lung cancer':ti,ab OR 'colorectal cancer':ti,ab OR 'colon cancer':ti,ab OR 'rectal cancer':ti,ab OR 'esophageal cancer':ti,ab OR 'gastric cancer':ti,ab OR 'liver cancer':ti,ab OR 'hepatocellular carcinoma':ti,ab OR 'pancreatic cancer':ti,ab OR 'cholangiocarcinoma':ti,ab OR 'gallbladder carcinoma':ti,ab) NOT ('hematologic disease'/exp OR 'multiple myeloma'/exp OR 'leukemia'/exp OR 'lymphoma'/exp OR 'leukemia':ti,ab OR 'myeloma':ti,ab OR 'lymphoma':ti,ab OR 'lymphoproliferative':ti,ab OR

'myeloid':ti OR 'myelodysplastic syndrome':ti,ab OR 'lymphoma*':ti OR 'hodgkin':ti,ab OR 'immunoproliferative small intestinal':ti,ab OR 'aml':ti OR 'cll':ti OR 'cml':ti OR 'apl':ti OR 'hl':ti OR 'nhl':ti OR 'hematopoietic*':ti OR 'haematologic*':ti) AND ('circulating tumor dna'/exp/mj OR 'circulating tumor dna':ab,ti OR 'ctdna':ab,ti OR 'ct dna':ab,ti OR 'minimal residual disease'/exp/mj OR 'molecular residual':ab,ti OR 'minimal residual':ab,ti OR 'mrd':ab,ti OR 'residual minimal':ab,ti OR 'measurable residual':ab,ti)) NOT ('chlamydiasis'/exp OR 'chlamydiasis':ab,ti OR 'chloroplast'/exp OR 'chloroplast':ab,ti OR 'chloroplast*':ab,ti OR 'etioplast*':ab,ti OR 'etioplast':ab,ti OR 'calf thymus':ab,ti) AND ([article]/lim OR [conference abstract]/lim OR [conference paper]/lim) AND [humans]/lim) NOT ('review':ti OR 'meta':ti) AND [2016-2022]/py AND [embase]/lim NOT [medline]/lim

3. Search strategy for Cochrane Library

#1	MeSH descriptor: [Neoplasms] explode all trees
#2	("breast cancer"):ti,ab,kw
#3	("lung cancer"):ti,ab,kw
#4	("colorectal cancer"):ti,ab,kw
#5	(colon cancer):ti,ab,kw
#6	(rectal cancer):ti,ab,kw
#7	("esophageal cancer"):ti,ab,kw
#8	("gastric cancer"):ti,ab,kw
#9	("liver cancer"):ti,ab,kw
#10	(hepatocellular carcinoma):ti,ab,kw
#11	("pancreatic cancer"):ti,ab,kw
#12	("cholangiocarcinoma"):ti,ab,kw
#13	(gallbladder carcinoma):ti,ab,kw
#14	#1 OR #2 OR #3 OR #4 OR #5 OR #6 OR #7 OR #8 OR #9 OR #10 OR #11 OR #12 OR #13
#15	MeSH descriptor: [Hematologic Neoplasms] explode all trees
#16	MeSH descriptor: [Multiple Myeloma] explode all trees
#17	MeSH descriptor: [Leukemia] explode all trees
#18	MeSH descriptor: [Lymphoma] explode all trees
#19	("leukemia"):ti,ab,kw
#20	("myeloma"):ti,ab,kw
#21	("lymphoma"):ti,ab,kw
#22	("lymphoproliferative"):ti,ab,kw
#23	("myeloid"):ti,ab,kw
#24	("myelodysplastic"):ti,ab,kw
#25	("lymphoma*"):ti,ab,kw
#26	("Hodgkin"):ti,ab,kw
#27	("Immunoproliferative Small Intestinal"):ti,ab,kw
#28	("AML"):ti
#29	("CLL"):ti
#30	("CML"):ti
#31	("APL"):ti
#32	("HL"):ti
#33	("NHL"):ti

#34	("hematopoietic*"):ti
#35	("haematologic*"):ti
#36	#15 OR #16 OR #17 OR #18 OR #19 OR #20 OR #21 OR #22 OR #23 OR #24 OR #25 OR #26 OR #27 OR #28 OR #29 OR #30 OR #31 OR #32 OR #33 OR #34 OR #35
#37	#14 NOT #36
#38	MeSH descriptor: [Circulating Tumor DNA] explode all trees
#39	("Circulating Tumor DNA"):ti,ab,kw
#40	("ctDNA"):ti,ab,kw
#41	("ct DNA"):ti,ab,kw
#42	("tumor dna circulating"):ti,ab,kw
#43	#38 OR #39 OR #40 OR #41 OR #42
#44	("molecular residual disease*"):ti,ab,kw
#45	("minimal residual"):ti,ab,kw
#46	("MRD"):ti
#47	("residual minimal"):ti,ab,kw
#48	("measurable residual"):ti,ab,kw
#49	#44 OR #45 OR #46 OR #47 OR #48
#50	#43 OR #49
#51	#37 AND #50
#52	#51 AND "2016-2022"

4. Search strategy for clinicaltrial.gov

("Circulating Tumor DNA" OR "ctDNA" OR "ct DNA" OR "molecular residual" OR "minimal residual" OR "MRD" OR "measurable residual" OR "residual minimal")|Studies With Results| ("Neoplasms") NOT ("hematologic neoplasms" OR " myeloma" OR "leukemia" OR "lymphoma" OR "lymphoproliferative" OR "myeloid" OR "myelodysplastic syndrome" OR "Hodgkin" OR "immunoproliferative small intestinal")

5. Search strategy for WHO-ICTRP

title	Circulating Tumor DNA OR ctdna OR ctDNA OR ct DNA OR circulating tumor DNA OR molecular residual disease OR minimal residual OR minimal residual disease OR minimal residual diseases OR residual minimal disease OR MRD OR measurable residual disease
condition	cancer OR tumor OR neoplasm OR carcinoma

6. Search strategy for WOS (the web of science)

((((((((((((TS=(Neoplasms)) OR TS=("breast cancer")) OR TS=("lung cancer")) OR TS=("colorectal cancer")) OR TS=("colon cancer")) OR TS=("rectal cancer")) OR TS=("esophageal cancer")) OR TS=("gastric cancer")) OR TS=("liver cancer")) OR TS=(" hepatocellular carcinoma ")) OR TS=("pancreatic cancer")) OR TS=("gallbladder carcinoma ")) OR TS=("cholangiocarcinoma") NOT (((((((((((((((((TS=(Hematologic neoplasms)) OR TS=(Myeloma)) OR TS=(Leukemia)) OR TS=(Lymphoma)) OR TS=("Myelodysplastic Syndromes")) OR AB=(lymphoproliferative)) OR TS=(myeloid)) OR TI=("lymphoma*")) OR TI=("Hodgkin")) OR TS=("Immunoproliferative Small Intestinal")) OR TI=("AML")) OR TI=("CLL")) OR TI=("CML")) OR TI=("APL")) OR TI=("HL")) OR TI=("NHL")) OR TI=("hematopoietic*")) OR TI=("haematologic*"))) AND (((((((TS=("Circulating Tumor DNA")) OR TS=("ctDNA")) OR TS=("molecular residual")) OR TS=("minimal residual")) OR TS=("MRD")) OR TS=("residual minimal")) OR TS=("measurable residual")) and Article or Abstract or Meeting (Document Types) and 2022 or 2021 or 2020 or 2019 or 2018 or 2017 or 2016 (Publication Years)

Supplementary Table 3 Exposure and outcome measurement

Study ID	Measure time of ctDNA	Positive definition of ctDNA detection	Outcome	Outcome Definition
Anandappa,2021	-	-	RFS\Recurrence	-
Beagan,2020	Preoperative blood samples were collected after patients were placed under general anesthesia but immediately before surgical incision for the intended CRS-HIPEC procedure; at least one postoperative blood sample was taken by venipuncture from all CRS-HIPEC patients, typically within 2–4 weeks, but no later than 3 months after the procedure; further samples were taken during routine follow-up every 3 months, up to 25 months after CRS-HIPEC. If a recurrence was diagnosed during follow-up by physical assessments and (PET-) CT imaging, an additional blood sample was taken at diagnosis or within 1 month	-	DFS\recurrence	-
Benešová,2019	before, 1 week after the surgery, and subsequently in several-month intervals during their follow-ups	-	Recurrence	-
Benhaim,2021	-	-	RFS\recurrence	-
Bidard,2019	Before starting treatment, after 1 month of systemic therapy (all patients), and before any surgical resection of LM (in patients referred to surgery after the shrinkage of LM)	Samples with a variant allele frequency <0.1% were classified as ctDNA-negative	OS	OS was defined as time from inclusion to death from any cause
Bolhuis,2021	Prior to systemic treatment (baseline), preoperatively, a maximum of 100 days postoperatively, and during	-	RFS\recurrence\pCR	RFS was calculated from the date of hepatic resection until

Study ID	Measure time of ctDNA	Positive definition of ctDNA detection	Outcome	Outcome Definition
	follow-up			documented progression or censored on the last clinical visit date. In the case of a two-stage hepatic resection, RFS was calculated from the last surgical procedure
Boysen,2020	The post-treatment blood sample for translational analysis was drawn 2 weeks after the local procedure. Further samples were taken, if available, before treatment and at a fixed schedule during follow up；For patients receiving adjuvant chemotherapy, blood samples were drawn during treatment.	-	RFS\recurrence	Both local and distant recurrences alongside death were considered as an event in the time to recurrence analysis.
Chan,2020	A preoperative blood sample was taken just before surgery and postoperative samples were collected on multiple time-points to monitor the changes of mutation profiles	-	lead time	-
Chee,2022	After curative intent surgery or radiation	-	RFS\recurrence\lead time	-
Chen,2021	Preoperatively within 7 days, postoperatively at day 3–7 before discharge, 6 months after surgery, and then every 3 months until month 24 unless the patient passed away or withdrew informed consent	A plasma sample was declared as ctDNA-positive if the number of true variants detected in the plasma was more than 5% of the number of total tracking variants in each patient	RFS\recurrence\lead time	RFS was calculated from the date of surgery to the date of verified radiological recurrence or death as a result of CRC for patients who relapsed and was censored at last follow up or non-CRC-related

Study ID	Measure time of ctDNA	Positive definition of ctDNA detection	Outcome	Outcome Definition
				death for patients who were not documented with recurrence
Ciardiello,2021	-	-	RFS\OS	-
Gu,2021	Blood samples were obtained before and 7-10 days after surgery	-	RFS	-
Henriksen,2022	Samples at diagnosis, postoperative, during adjuvant therapy and routine follow-up; Postoperative plasma samples collected before initiation of ACT were available for 140 patients	A previously validated cutoff of≥2 variants detectedwas used as criteriafor ctDNA positivity	RFS\recurrence\lead time	RFS was assessed by standard radiologic criteria and measured from date of surgery to verified first radiologic recurrence (local or distant)
Henriksen,2021	After surgery	-	RFS\lead time	-
Huang,2019	Prior to surgery, postoperative plasma samples were collected successively one month (1st), two months (2nd),and half a year (3rd) after surgery	a mutation was called if > 4 mutant reads were found in plasma with >= 1 read on each strand.	RFS\recurrence	-
Jin,2021	Preoperative blood samples were collected before surgery. Postoperative blood samples were collected within 2 weeks (1 to 14 d) after surgery. Additional blood samples were collected at follow-up	A plasma sample was deemed positive if ΔCq (VICmean − FAMmean) >−1 and negative if ΔCq (VICmean − FAMmean) ≤−1. VICmean and FAMmean values were calculated from the two duplicate reactions of each sample.	RFS\recurrence\lead time	-
Khakoo,2020	Serial blood samples were collected: pretreatment (within 4 weeks prior to commencing CRT), mid-CRT (3–4 weeks from the start of CRT), after completion of CRT (4–12 weeks from completion), and post-surgery (within 4–12 weeks)	a plasma was designated positive if a minimum of two mutant-positive droplets were present for at least one variant	DFS\MFS\OS\recurrence	DFS was measured from date of surgery until relapse or death from any cause and was censored at the last follow-up.

Study ID	Measure time of ctDNA	Positive definition of ctDNA detection	Outcome	Outcome Definition
Knudsen,2021	MFS was measured from study entry to development of metastases or death from any cause and was censored at the last follow-up.			
Kobayashi,2021	OS was measured from study entry to death from any cause or censored by last follow-up if alive.			
Kotaka,2021	"			
Lee,2021	-	-	Recurrence	-
Lim,2022	Preoperative plasma samples obtained within a month of hepatectomy	detects single nucleotide variants, indels, fusions, and copy number alterations in 74 genes with a reportable range ≥0.04%, ≥0.02%,≥0.04%, and ≥ 2.12 copies, respectively, and microsatellite instability (MSI). If more than one somatic genomic alteration was detected in the plasma, the plasma was considered positive for ctDNA	RFS\recurrence\OS	RFS was defined as the time from the date of hepatectomy to the date of the first radiological recurrence of the disease or the date of death due to any cause.
Liu,2022	-	-	DFS	-
Loupakis,2021	postoperative	-	RFS\recurrence	-
McDuff,2021	Serial blood samples were obtained from mCRC patients before and during first-line palliative chemotherapy at fixed intervals (after every four cycles) until confirmed disease progression	-	lead time	-
McNamara,2022	-	We classified the ctDNA fraction as positive or negative with a cut-off of 0.05% in further analyses to avoid false-positive MRD detection from background noise mutations	RFS\OS\recurrence\pCR\lead time	Relapse-free survival (RFS) was measured from the date of randomization to the first occurrence of local-regional failure or distant

Study ID	Measure time of ctDNA	Positive definition of ctDNA detection	Outcome	Outcome Definition
				metastasis
Murahashi ,2020	Plasma collected after surgery, before the start of ACT, was available for 112 patients	Plasma samples with ≥ 2 SNVs detected above a predefined confidence threshold were deemed ctDNA-positive	DFS\OS\recurrence\lead time	The primary objective was to measure the DFS from time of surgery to the first radiologic evidence of disease progression
Murray,2018	Plasma ctDNA was collected at baseline prior to neoadjuvant CRT, preoperatively, and postoperatively. Preoperative ctDNA assessments were performed between 0 and 17 weeks preoperatively. Postoperative ctDNA assessments were performed between 1 and 5 months postoperatively	detectable (≥ 2 mutant ctDNA alleles among a minimum of 2,000 wild-type alleles) ; if only one mutant ctDNA allele was detected, the patient's ctDNA status was classified as negative.	Recurrence\pCR	-
Ng,2017	Drawn 1 to 34 days after R0-resection	If a tissue baseline variant was detected in cfDNA with a significant adjusted p-value, the patient was defined ctDNA+, and ctDNA- otherwise	Recurrence	-
Nishioka,2022	Before the initiation of preoperative therapy (baseline), after preoperative treatment (post treatment, just before surgery) and at 12 weeks after surgery (post operation)	The cut-off value for the mutant allele fraction (MAF) was 0.15%	RFS\recurrence\pCR	-
Øgaard,2021	ctDNA status was determined within 12 months after surgical therapy for the primary cancer; Samples were collected within 2.3 (median, IQR 1.6–7.3) months post-surgery	A sample was deemed ctDNA positive if at least one PCR replicate was positive for either methylated BCAT1 or IKZF1	RFS\OS\recurrence	-
Parikh,2021	-	-	Recurrence	-
Peng,2020	Postoperative	-	RFS\recurrence	-

Study ID	Measure time of ctDNA	Positive definition of ctDNA detection	Outcome	Outcome Definition
Schøler,2017	Sample prior to liver resection (termed pre-OP), a postoperative sample collected within 3 months after resection (termed postOP), and serial samples collected every third month up to 36 months after resection. For patients administered ACT, the first blood sample collected after the end of therapy was termed post-ACT. For all patients, we defined 'an end of definitive treatment (EOT)' sample.	Samples were classified as 'TriMeth positive' if two out of three TriMeth markers showed >1 positive droplet; otherwise, they were classified as 'TriMeth negative', as described by Jensen et al	RFS\recurrence\lead time	-
Sefrioui,2021	-	-	RFS\recurrence	RFS was measured from the day of completion of definitive treatment to first radiographic recurrence or death from colorectal cancer
Suzuki,2020	The plasmas were collected 3-5 weeks after surgery and after last cycle of ACT(Adjuvant chemotherapy）	Patients were classified as ctDNA (+) or (-) based on the detection of SNVs identified in tumor tissue at an AF of at least 5%	RFS	-
Taieb,2021	Blood samples were collected at day 0 (pre-op), 8, 30, and every three months until death, patient withdrawal from the study, or month 36	-	RFS\OS\recurrence\lead time	-
Tarazona,2019	-	-	RFS\OS\recurrence	The PFS was defined as the time from inclusion to disease progression or death from any cause, whichever occurred first.
Tarazona,2020	The OS was defined as the time from inclusion to death from any cause"			
Thomsen,2020	Peripheral blood at multiple time	detection of mutated ctDNA (at least	RFS	-

Study ID	Measure time of ctDNA	Positive definition of ctDNA detection	Outcome	Outcome Definition
	points including pre-operation, post-operation (average 7.3 days after operation), and every 3 months follow-up – aiming to collect serially until 2 years	2 copies) in the sample of "post-operation"		
Tie,2019	-	The average fractions of methylated genes was calculated as well as standard deviation of the data and used to determine a "threshold of positivity" equivalent to "averageþ2SD" (0.3% and 0.21% for NPY and WIF1, respectively)；The obtained percentage of MetctDNA was compared with the "threshold of positivity" determined as described above. All results above this threshold were considered positive and the remaining ones as negative；	DFS\OS\recurrence	Disease free survival (DFS) measured from the date of randomization to the first documented radiological recurrence, second CC, or death, whichever occurred first.
Tie,2019	Overall survival (OS) defined as the time from randomization to the date of death from any cause."			
Tie,2016	Blood samples for ctDNA analysis were collected at baseline, 6-8 weeks after surgery, and every 4 months for up to 5 years	-	DFS\recurrence\lead time	DFS according to standard radiological criteria
Tsukada,2022	-	-	RFS\recurrence\lead time	-
Unseld,2021	-	Tumor-specific methylated DNA was defined as DNA with methylation of the NPY gene promoter；The meth-ctDNA level was defined as the fraction of meth-ctDNA and expressed as the	PFS\OS	Progression-free survival (PFS) was calculated to the date of progression, death, or censored at last hospital contact.

Study ID	Measure time of ctDNA	Positive definition of ctDNA detection	Outcome	Outcome Definition
		proportion of NPY methylated alleles in the total circulating cell-free DNA；zero-negative; elevated-positive		
Vidal,2021	Overall survival (OS) was calculated to the date of death from any cause."			
Wang,2021	Blood samples for ctDNA analysis were collected 4 to10 weeks after surgery (before commencing any adjuvant chemotherapy) and at completion of treatment (within 6 weeks of the final cycle of chemotherapy)			
Wang,2019				
Zhou,2021		For each patient, 1 mutation identified in the tumor tissue was assessed in the plasma for the presence of ctDNA；ctDNA was classified as detectable (ctDNA-positive) or undetectable (ctDNA negative) based on a permutation test that compared the mutation frequency in the sample of interest with the mutation frequency in controls.	RFS\recurrence	Recurrence-free interval (RFI), measured from the date of surgery to documented first radiological recurrence or death as a result of colorectal cancer, and was censored at last follow-up or non-colorectal cancer related death
Appelt,2019	Plasma samples were collected pretreatment, postchemoradiotherapy and 4–10 weeks after surgery	ctDNA was classified as detectable (ctDNA positive) or undetectable (ctDNA negative) based on a permutation test that compared the mutation frequency in the sample of interest with the mutation frequencies in controls。the MAF, defined as the ratio between the	RFS\recurrence\pCR	The primary outcome measure was recurrence-free survival (RFS), measured from date of surgery to documented first recurrence or death as a result of colorectal

Study ID	Measure time of ctDNA	Positive definition of ctDNA detection	Outcome	Outcome Definition
		number of supermutants and the number of UIDs for the mutation of interest, was calculated for each well with >200 UIDs. The difference in the distributions of the MAFs between the sample of interest and the controls was then statistically evaluated via an exact permutation test, using the permTS function of the R perm package。A P value of 0.1 was then chosen as the significance threshold to classify a sample of interest as ctDNA positive (P<0.1) or ctDNA negative		cancer, and was censored at last follow-up or non-colorectal cancer-related death.
Elez,2019	Pathology reports from resection specimens were reviewed to assess tumour regression following chemoradiotherapy, with pCR defined as ypT0N0."			
Hamfjord,2021	-	ctDNA was classified as detectable (ctDNA-positive) or undetectable (ctDNA-negative) on the basis of a permutation test that compared the mutation frequency in the sample of interest with the mutation frequencies in controls；A 0.1 P value was then chosen as the threshold to classify a sample of interest as ctDNA-positive (P < 0.1) or ctDNA-negative.	RFS\recurrence\lead time	RFS was measured from date of surgery to documented first radiologic recurrence or death as a result of colorectal cancer and was censored at last follow-up or non–colorectal cancer–related death.
Ji,2021	-	-	RFS\recurrence	-
Wang,2021	Prior to initiation of induction chemotherapy with mFOLFOX6	-	DFS\OS\pCR	DFS was defined as the time from randomization

Study ID	Measure time of ctDNA	Positive definition of ctDNA detection	Outcome	Outcome Definition
	þ/aflibercept (baseline), and after completing all TNTs within 48 hours before surgery (presurgery).			until recurrence, second primary tumor, or death, whichever occurred first, independently of whether patient underwent surgery or not.
Li,2022	The primary endpoint of the main study was the pCR rate defined as the absence of viable tumor cells in the primary tumor and lymph nodes (ypT0ypN0).			
Reinert,2022	OS was defined as the time from randomization to death from any cause.			
Tie,2021				
Mason,2021	Blood samples were collected at diagnosis before treatment (baseline), before liver resection (preoperation), after liver resection (post-operation), after completion of post-operative adjuvant chemotherapy(post-ACT) and in the event of disease progression(PD).	A sample was defined as positive when variant allele frequency (VAF) ≥ 2% for WES Plus and ≥ 0.5% for the ctDNA 451-gene panel	RFS\recurrence	RFS was the time interval from the date of liver resection to disease recurrence or the last date of follow-up.
Gao,2023	Blood samples from participants were collected at 1 month after the surgical procedure and then every 3 to 6 months thereafter for ctDNA level analysis.	The method incorporated the following steps. First, the mutant allele fraction (MAF), defined as the ratio between the number of supermutants and the number of UIDs for the mutation of interest, was calculated for each well with >200 UIDs. The difference in the distributions of the MAFs	Recurrence\lead time	-

Study ID	Measure time of ctDNA	Positive definition of ctDNA detection	Outcome	Outcome Definition
		between the sample of interest and the experiment-specific controls was then statistically evaluated with the permutation test, using the permTS function of the R package perm (R software version 3.2.3). A one-sided test rather than a two-sided test was used to avoid attributing significance to a ctDNA-negative sample that has fewer supermutants than the associated control. A P-value of 0.02 was chosen as the threshold to classify a sample of interest as ctDNA-positive (P < 0.02) or ctDNA-negative (P >0.02). Given the lack of a gold standard, the choice of this specific P-value threshold (P < 0.02) was motivated by the fact that a specificity of at least 98% was considered desirable		
Kotani,2023	Peripheral blood samples were collected at four time points, before nCRT (Baseline), one cycle after the initiation of nCRT (On-nCRT), 174 about seven weeks after nCRT and before surgery (Pre-op), and within one month after 175 surgery (Post-op)	ctDNA positivity was defined as when at least one mutation in a tissue sample had also been detected in matched ctDNA	MFS\pCR	-
Grancher,2022	-	Samples were considered positive for meth-ctDNA if >2 positive droplets/sample, and fractional abundance of meth-ctDNA was calculated as proportion of meth-ctDNA relative to total amount of	MFS\OS	For estimation of OS, death from any cause was considered an event, and patients were censored if alive at the time of data update

Study ID	Measure time of ctDNA	Positive definition of ctDNA detection	Outcome	Outcome Definition
		circulating DNA		
Reichert,2023	Before first- and/or second-line treatment	Mutant allele fractions were calculated as the number of mutant beads divided by the total number of beads analyzed, and all samples were analyzed blinded to the study endpoints;MAF>5.8%	RFS\OS	PFS was defined as the time from treatment start to disease progression or death.
Reinert,2019	OS was defined as the time from mCRC diagnosis to death from any cause or the last follow-up visit"			
Symonds,2021	Blood was collected at baseline prior to first-line therapy.	A sample was scored as positive if the number of positive events is greater than or equal to the call cut-off for the respective assay and the minor Allele frequency (MAF) ≥0.1%； A sample was scored as negative if the number of positive events is lessthan the call cut-off for the respective assay or the MAF <0.1%	RFS\OS	-
Tie,2022	Blood samples were collected at baseline, after nCRT, and 1 day after operation.	TMB in ctDNA>10 Mb	Recurrence\pCR	-

Abbreviation: RFS, recurrence-free survival; OS, overall survival; pCR, pathological complete response; “-”, not reported.

Supplementary Table 4 Risk of Bias
Table 4.1 Newcastle-Ottawa scale for observational study

ID	Selection				Comparability	Outcome			Score
	Representativeness of the exposed cohort	Selection of the non exposed cohort	Ascertainment of exposure	Demonstration that outcome of interest was not present at start of study	Comparability of cohorts on the basis of the design or analysis	Assessment of outcome	Was follow-up long enough for outcomes to occur	Adequacy of follow up of cohorts	
Anandappa,2021	0	0	1	1	1	0	1	1	5
Beagan,2020	1	1	1	1	1	0	1	1	7
Benešová,2019	1	1	1	1	1	0	1	1	7
Benhaim,2021	1	1		1	2	0	1	1	7
Bidard,2019	0	0	1	1	1	1	1	1	6
Bolhuis,2021	1	1	1	1	2	1	1	1	9
Boysen,2020	1	1	1	1	2	1	1	1	9
Chan,2020	1	1	0	0	0	0	1		3
Chee,2022	1	1	0	1	0	0	1	1	5
Chen,2021	1	1	1	1	2	1	1	1	9
Ciardiello,2021	0	0	0	0	2	0	0	1	3
Gu,2021	1	1	1	1	1	0	0	0	5
Henriksen,2022	1	1	1	1	2	1	1	1	9
Henriksen,2021	0	0	1	1	0	0	1	1	4
Huang,2019	0	0	1	1	1	0	1	1	5
Jin,2021	0	0	1	1	1	0	1	1	5

ID	Selection				Comparability	Outcome			Score
	Representativeness of the exposed cohort	Selection of the non exposed cohort	Ascertainment of exposure	Demonstration that outcome of interest was not present at start of study	Comparability of cohorts on the basis of the design or analysis	Assessment of outcome	Was follow-up long enough for outcomes to occur	Adequacy of follow up of cohorts	
Khakoo,2020	0	0	1	1	1	1	1	1	6
Knudsen,2021	0	0	0	1	0	0	1	1	3
Kobayashi,2021	1	1	1	1	1	1	1	1	8
Kotaka,2021	1	1	0	0	0	0	1	0	3
Lee,2021	0	0	1	1	1	0	1	1	5
Lim,2022	1	1	0	0	0	0	1	0	3
Liu,2022	0	0	1	1	2	1	1	1	7
Loupakis,2021	0	0	1	1	2	1	1	1	7
McDuff,2021	0	0	1	1	1	1	1	1	6
McNamara,2022	0	0	1	1	1	0	0	1	4
Murahashi ,2020	0	0	1	1	2	0	0	1	5
Murray,2018	0	0	1	1	1	0	1	1	5
Ng,2017	1	1	1	1	1	0	1	1	7
Nishioka,2022	1	1	0	1	2	0	1	1	7
Øgaard,2021	1	1	1	1	1	0	1	1	7
Parikh,2021	1	1	1	1	1	1	1	1	8
Peng,2020	0	1	1	1	1	0	0	1	5
Schøler,2017	1	1	1	1	0	1	1	1	7

ID	Selection				Comparability	Outcome			Score
	Representativeness of the exposed cohort	Selection of the non exposed cohort	Ascertainment of exposure	Demonstration that outcome of interest was not present at start of study	Comparability of cohorts on the basis of the design or analysis	Assessment of outcome	Was follow-up long enough for outcomes to occur	Adequacy of follow up of cohorts	
Sefrioui,2021	0	0	0	1	2	1	0	1	5
Suzuki,2020	1	1	1	1	0	0	0	0	4
Taieb,2021	1	1	1	1	2	1	1	1	9
Tarazona,2019	1	1	1	1	2	0	1	1	8
Tarazona,2020	0	0	1	1	1	0	1	0	4
Thomsen,2020	1	1	1	1	1	1	1	1	8
Tie,2019	1	1	1	1	2	1	1	1	9
Tie,2019	1	1	1	1	2	1	1	1	9
Tie,2016	1	1	1	1	2	1	1	1	9
Tsukada,2022	1	1	0	1	1	0	1	1	6
Unseld,2021	1	1	1	1	1	0	1	0	6
Vidal,2021	1	1	0	1	1	1	1	1	7
Wang,2021	1	1	1	1	1	1	1	1	8
Wang,2019	1	1	1	1	1	1	1	1	8
Zhou,2021	1	1	1	1	1	0	1	1	7
Appelt,2019	0	1	1	1	1	1	1	1	7
Elez,2019	0	0	1	1	1	1	0	1	5
Hamfjord,2021	1	1	1	1	1	0	1	1	7

ID	Selection				Comparability	Outcome			Score
	Representativeness of the exposed cohort	Selection of the non exposed cohort	Ascertainment of exposure	Demonstration that outcome of interest was not present at start of study	Comparability of cohorts on the basis of the design or analysis	Assessment of outcome	Was follow-up long enough for outcomes to occur	Adequacy of follow up of cohorts	
Ji,2021	0	0	0	1	1	1	1	0	4
Wang,2021	1	1	0	1	1	1	0	0	5
Li,2022	1	1	1	2	1	1	1	1	9
Reinert,2022	1	1	1	1	1	1	1	1	8
Tie,2021	1	1	1	1	2	1	1	1	9
Mason,2021	1	1	1	1	1	0	1	1	7
Gao,2023	1	1	1	1	1	1	1	1	8
Kotani,2023	1	1	1	1	2	1	1	1	9
Grancher,2022	1	1	1	1	2	1	1	1	9
Reichert,2023	1	1	1	1	1	0	0	0	5
Reinert,2019	1	1	1	1	2	1	1	1	9
Symonds,2021	1	1	0	1	1	0	1	1	6
Wong,2019	1	1	1	1	0	1	1	1	7

Supplementary Table 5 Subgroup analysis

Outcome	Timepoint	Subgroup		Number of studies	Effect estimates		I^2	*P* value	*P* value subgroup difference
					HR	95%CI			
RFS	Baseline	Cancer type	CRC	4	2.43	1.07-5.50	*53.68%*	0.033	0.767
			LARC	4	1.83	1.13-2.94	*0.00%*	0.014	
			mCRC	9	2.19	1.65-2.90	*14.91%*	<0.001	
		Tumor-inform	Y	7	1.96	1.21-3.17	*27.30%*	0.006	0.571
			N	10	2.30	1.74-2.04	*10.83%*	<0.001	
		Resectable	Y	14	2.34	1.80-3.03	*11.20%*	<0.001	0.096
			N	3	1.72	1.34-2.20	*0.00%*	<0.001	
	During NAT	Tumor-inform	Y	2	4.82	1.52-15.35	*0.00%*	0.008	0.448
			N	1	2.60	0.87-7.80	-	0.088	
	After NAT and before surgery	Tumor-inform	Y	4	11.57	5.81-23.07	*23.33%*	<0.001	0.209
			N	2	5.71	2.42-13.51	*0.00%*	<0.001	
	After surgery	Cancer type	CRC	22	6.97	4.99-9.73	*83.30%*	<0.001	**0.0015**
			LARC	3	13.99	7.42-26.36	*0.00%*	<0.001	
			mCRC	8	4.24	3.17-5.68	*11.03%*	<0.001	
		Tumor-inform	Y	22	8.49	7.02-10.27	*9.36%*	<0.001	**<0.001**
			N	11	3.10	2.15-4.46	*59.81%*	<0.001	
	After surgery and during ACT	Cancer type	LARC	2	23.53	7.70-71.87	*23.53%*	<0.001	**0.019**
			mCRC	1	3.36	1.03-10.95	*3.36%*	0.044	
		Cancer type	CRC	17	11.38	7.35-17.62	*49.15%*	<0.001	0.149
			mCRC	5	4.88	2.35-10.14	*79.96%*	<0.001	

			CRC/mCRC	1	9.70	2.50-37.62	-	<0.001	
	After full-course treatment	Tumor-inform	Y	14	11.20	6.49-19.32	*82.12%*	<0.001	0.063
			N	9	5.77	3.73-8.93	*4.11%*	<0.001	
		Resectable	Y	20	10.04	6.69-15.06	*58.48%*	<0.001	**<0.001**
			N	1	2.08	1.37-3.16	-	<0.001	
			mixed	2	8.42	3.62-19.59	*0.00%*	<0.001	
	Long-term post-treatment surveillance	Cancer type	CRC	7	19.03	9.09-39.85	*64.80%*	<0.001	0.172
			mCRC	3	8.91	4.00-19.84	*73.09%*	<0.001	
		Tumor-inform	Y	7	19.25	9.00-41.15	*77.70%*	<0.001	0.136
			N	3	8.85	4.47-17.50	*34.62%*	<0.001	

	**Timepoint**	**Subgroup**		**Number of studies**	**HR**	**95%CI**	***I*²**	***P* value**	***P* value subgroup difference**
OS	Baseline	Cancer type	LARC	2	2.09	1.26-3.48	*0.00%*	0.004	0.796
			mCRC	8	1.91	1.20-3.05	*70.59%*	0.007	
		Tumor-inform	Y	2	2.11	1.36-3.26	*0.00%*	0.001	0.818
			N	8	1.95	1.20-3.16	*70.11%*	0.007	
		Resectable	Y	5	1.41	0.71-2.81	*76.62%*	0.329	0.211
			N	5	2.24	1.80-2.77	*0.00%*	<0.001	
	After NAT and before surgery	Tumor-inform	Y	1	17.78	1.94-162.77	-	0.011	0.534
			N	2	7.00	1.02-48.15	*53.70%*	0.048	
	After surgery	Cancer type	CRC	2	2.72	0.68-10.96	*72.96%*	0.158	0.300
			mCRC	2	7.45	2.04-27.27	*54.80%*	0.002	
		Tumor-inform	Y	3	6.82	3.10-15.01	*9.61%*	<0.001	**0.001**
			N	1	1.56	1.08-2.26	-	0.018	

	After surgery and during ACT	Cancer type	LARC	1	6.10	0.39-95.03	-	0.197	0.959
			mCRC	1	6.60	2.07-21.05	-	0.001	
		Tumor-inform	Y	1	6.60	2.07-21.05	-	0.001	0.959
			N	1	6.10	0.39-95.03	-	0.197	
	After full-course treatment	Cancer type	CRC	2	3.70	1.50-9.10	*32.33%*	0.004	0.714
			mCRC	2	2.89	1.11-7.55	*65.92%*	0.03	
		Tumor-inform	Y	3	2.50	1.53-4.08	*34.13%*	<0.001	0.154
			N	1	6.60	1.90-22.86	-	0.003	
		Resectable	Y	3	4.06	2.16-7.64	*0.00%*	<0.001	0.056
			N	1	2.00	1.39-2.87	-	<0.001	

	**Timepoint**	**Subgroup**		**Number of studies**	**RR**	**95%CI**	***I*²**	***P* value**	***P* value subgroup difference**
Recurrence	Baseline	Cancer type	CRC	3	2.40	1.35-4.24	*0.00%*	0.003	0.288
			LARC	5	1.29	0.68-2.43	*14.14%*	0.431	
			mCRC	3	2.26	1.44-3.54	*0.00%*	<0.001	
		Tumor-inform	Y	4	1.86	1.24-2.79	*0.00%*	0.003	0.555
			N	7	2.24	1.39-3.62	*9.37%*	0.001	
		Resectable	Y	10	1.94	1.37-2.73	*0.00%*	<0.001	0.622
			N	1	2.36	1.16-4.81	-	0.018	
	During NAT	Tumor-inform	Y	1	3.18	0.74-13.63	-	0.120	0.653
			N	1	2.11	0.77-5.81	-	0.146	
	After NAT and before surgery	Tumor-inform	Y	3	5.58	2.68-11.63	*34.75%*	<0.001	0.482
			N	3	3.92	2.02-7.58	*0.00%*	<0.001	

	After surgery	Cancer type	CRC	20	4.13	3.26-5.25	*76.36%*	<0.001	**<0.001**
			LARC	2	6.09	3.33-11.16	*0.00%*	<0.001	
			mCRC	9	1.85	1.65-2.08	*0.00%*	<0.001	
		Tumor-inform	Y	20	3.50	2.72-4.52	*84.10%*	<0.001	**<0.001**
			N	11	1.98	1.73-2.27	*46.49%*	<0.001	
	After surgery and during ACT	Cancer type	LARC	3	5.12	1.04-25.29	*69.23%*	0.045	0.115
			mCRC	2	1.33	0.82-2.18	*55.57%*	0.249	
		Tumor-inform	Y	1	6.20	1.99-19.29	-	0.002	**0.026**
			N	4	1.52	0.93-2.49	*65.04%*	0.092	
	After full-course treatment	Cancer type	CRC	10	4.73	2.90-7.71	*79.15%*	<0.001	0.358
			mCRC	3	3.34	1.91-5.85	*54.53%*	<0.001	
		Tumor-inform	Y	8	5.26	2.95-9.36	*82.92%*	<0.001	0.126
			N	5	3.09	2.16-4.43	*34.32%*	<0.001	
		Resectable	Y	11	4.49	2.73-6.45	*78.37%*	<0.001	0.531
			mixed	2	5.47	2.68-11.15	*0.00%*	<0.001	
	Long-term post-treatment surveillance	Cancer type	CRC	4	17.45	5.51-55.22	*75.97%*	<0.001	**0.001**
			mCRC	4	2.29	1.83-2.86	*15.32%*	<0.001	
		Tumor-inform	Y	7	6.51	2.42-17.56	*84.88%*	<0.001	0.668
			N	1	5.05	2.75-9.26	-	<0.001	

	**Timepoint**	**Subgroup**		**Number of studies**	**OR**	**95%CI**	***I*²**	***P* value**	***P* value subgroup difference**
**pCR**	Baseline	Tumor-inform	Y	3	0.58	0.32-1.06	*0.00%*	0.077	0.492
			N	3	1.28	0.58-2.80	*67.64%*	0.910	

After NAT and before surgery	Tumor-inform	Y	3	0.34	0.09-1.33	*0.00%*	0.123	0.366
		N	4	0.76	0.26-2.21	*0.30%*	0.617	

Abbreviation: HR, hazard ratio; RR, risk ratio; OR, odds ratio; CRC, colorectal cancer; mCRC, metastatic colorectal cancer; LARC: localized advanced rectal cancer; CI, confidence interval; RFS, recurrence-free survival; OS, overall survival; pCR, pathologic complete response; NAT, neoadjuvant chemotherapy; ACT, adjuvant chemotherapy; Y, yes; N, no; -, not applicable.

Note: Baseline: before any treatment, for all CRCs; During NAT: only for LARC; After NAT and before surgery: only for LARC; After surgery: only for resectable CRCs; After surgery and during ACT: only for resectable and CRCs required ACT; After full-course treatment: after completing corresponding treatments, according to the clinical guidelines, for all CRCs; Long-term post-treatment surveillance: for all CRCs.

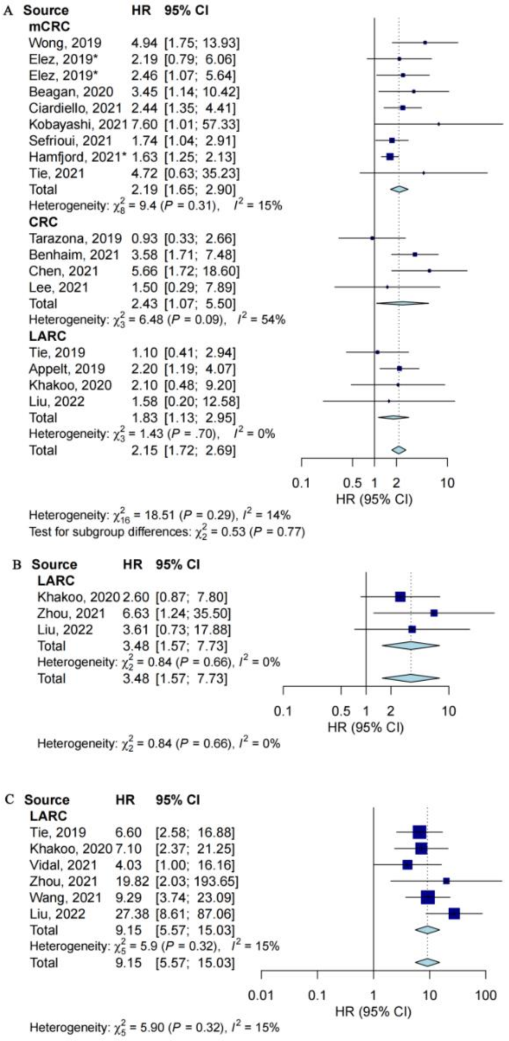

Supplementary Fig. 1 Forest plot of the association between ctDNA detection and RFS before treatment.

(A) at baseline (before any treatment, all CRCs); (B) during NAT (only for LARC); (C) after NAT and before surgery (only for LARC).

Abbreviation: CRC, colorectal cancer; CI, confidence interval; HR, hazard ratio; LARC: localized advanced rectal cancer; mCRC, metastatic colorectal cancer; NAT, neoadjuvant chemotherapy; RFS, recurrence-free survival.

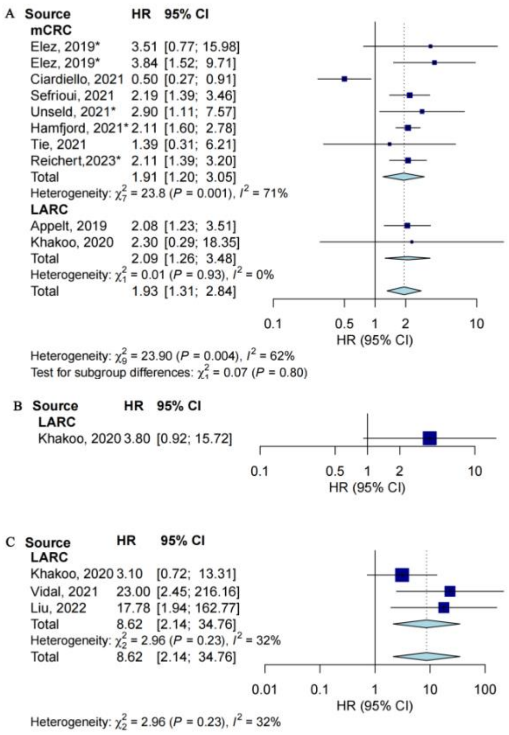

Supplementary Fig. 2 Forest plot of the association between ctDNA detection and OS before treatment.

(A) at baseline (before any treatment, all CRCs); (B) during NAT (only for LARC); (C) after NAT and before surgery (only for LARC).

Abbreviation: CRC, colorectal cancer; CI, confidence interval; HR, hazard ratio; LARC: localized advanced rectal cancer; mCRC, metastatic colorectal cancer; NAT, neoadjuvant chemotherapy; OS, overall survival.

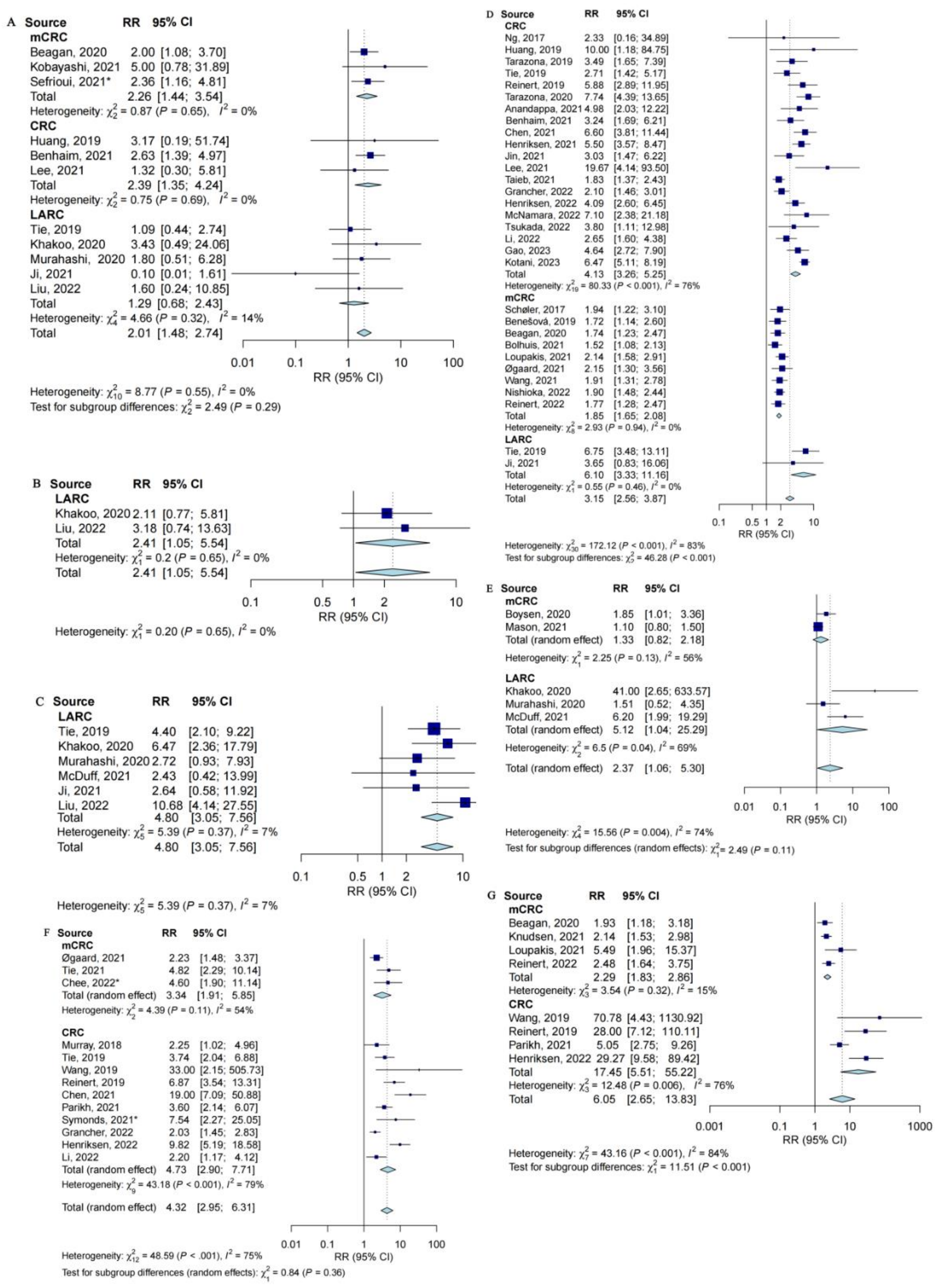

Supplementary Fig. 3. Forest plot of the association between ctDNA detection and recurrence.

(A) at baseline (before any treatment, all CRCs); (B) during NAT (only for LARC); (C) after NAT and before surgery (only for LARC); (D) just after surgery (only for resectable CRCs); (E) during surgery and ACT (only for resectable and CRCs required ACT); (F) after full-course treatment (after completing corresponding treatments according to the clinical guidelines, all CRCs); (G) long-term post-treatment surveillance (all CRCs).

Abbreviation: ACT, adjuvant chemotherapy; CRC, colorectal cancer; CI, confidence interval; LARC: localized advanced rectal cancer; mCRC, metastatic colorectal cancer; NAT, neoadjuvant chemotherapy; RR, risk ratio.

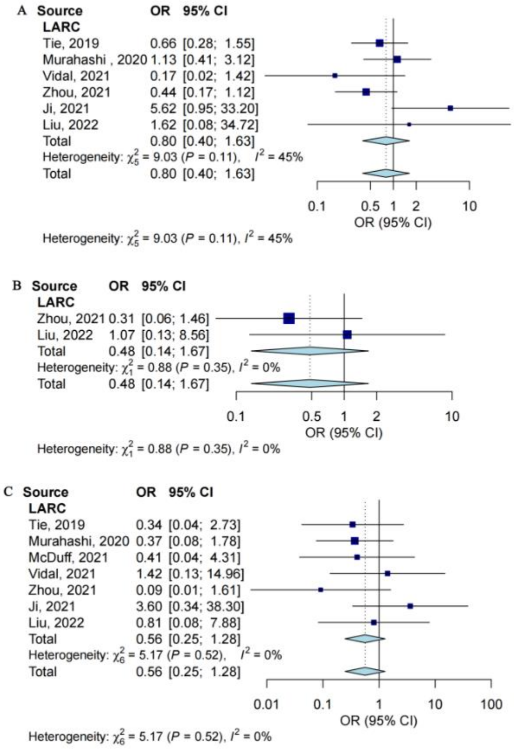

Supplementary Fig. 4. Forest plot of the association between ctDNA detection and pCR.

(A) at baseline (before any treatment, all CRCs); (B) during NAT (only for LARC); (C) after NAT and before surgery (only for LARC).

Abbreviation: CI, confidence interval; CRC, colorectal cancer; LARC, localized advanced rectal cancer; NAT, neoadjuvant chemotherapy; OR, odds ratio; pCR, pathologic complete response.

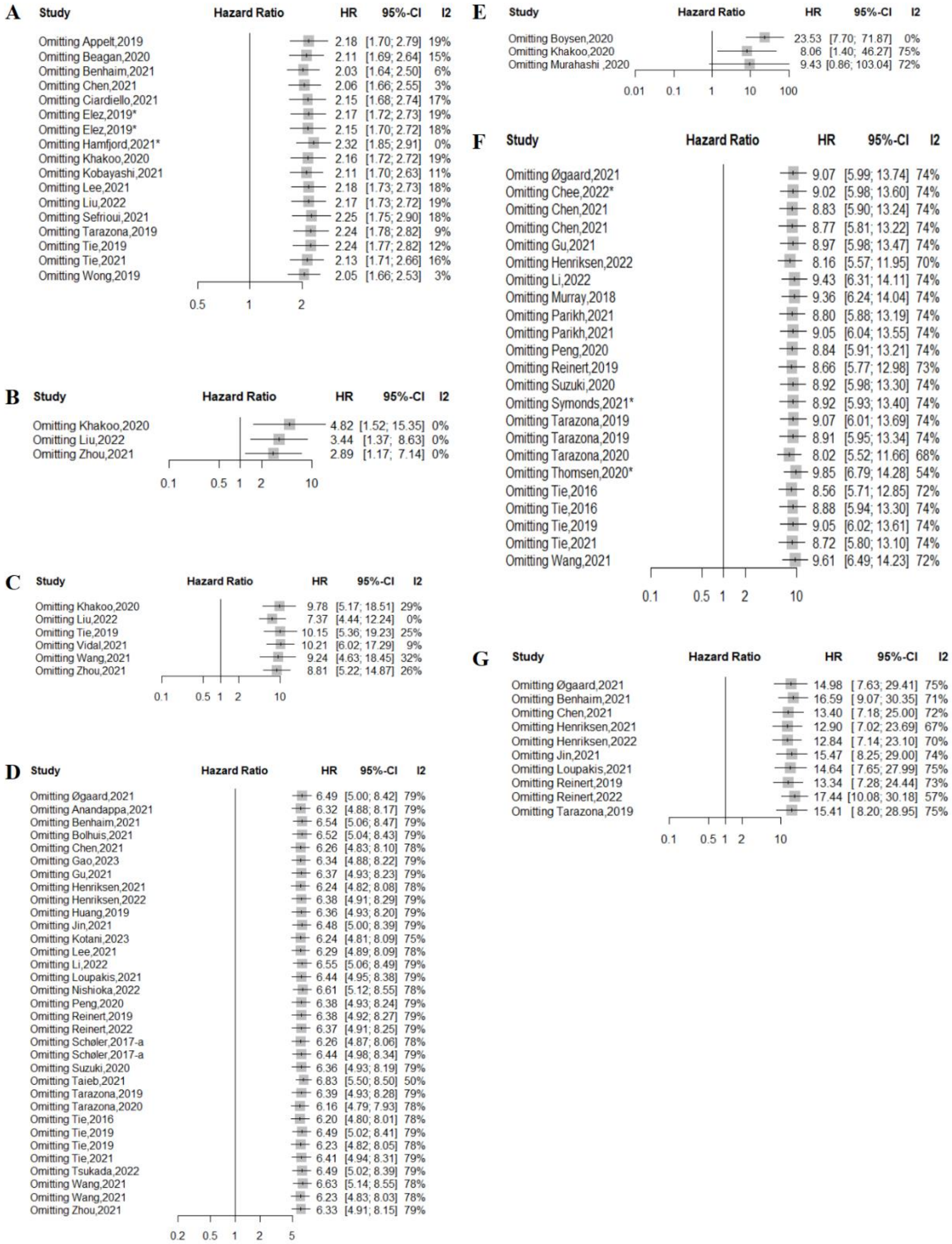

Supplementary Fig. 5 Sensitivity analysis of the association between ctDNA detection and RFS using leave-one-out methods.

(A) baseline (before any treatment, all CRCs); (B) during NAT (only for LARC); (C) after NAT and before surgery (only for LARC); (D) after surgery (only for resectable CRCs); (E) during surgery and ACT (only for resectable and CRCs required ACT); (F) after full-course treatment (after completing corresponding treatments according to the clinical guidelines, all CRCs); (G) long-term post-treatment surveillance (all CRCs).

Abbreviation: ACT, adjuvant chemotherapy; CRC, colorectal cancer; CI, confidence interval; HR, hazard ratio; LARC: localized advanced rectal cancer; mCRC, metastatic colorectal cancer; NAT, neoadjuvant chemotherapy; RFS, recurrence-free survival.

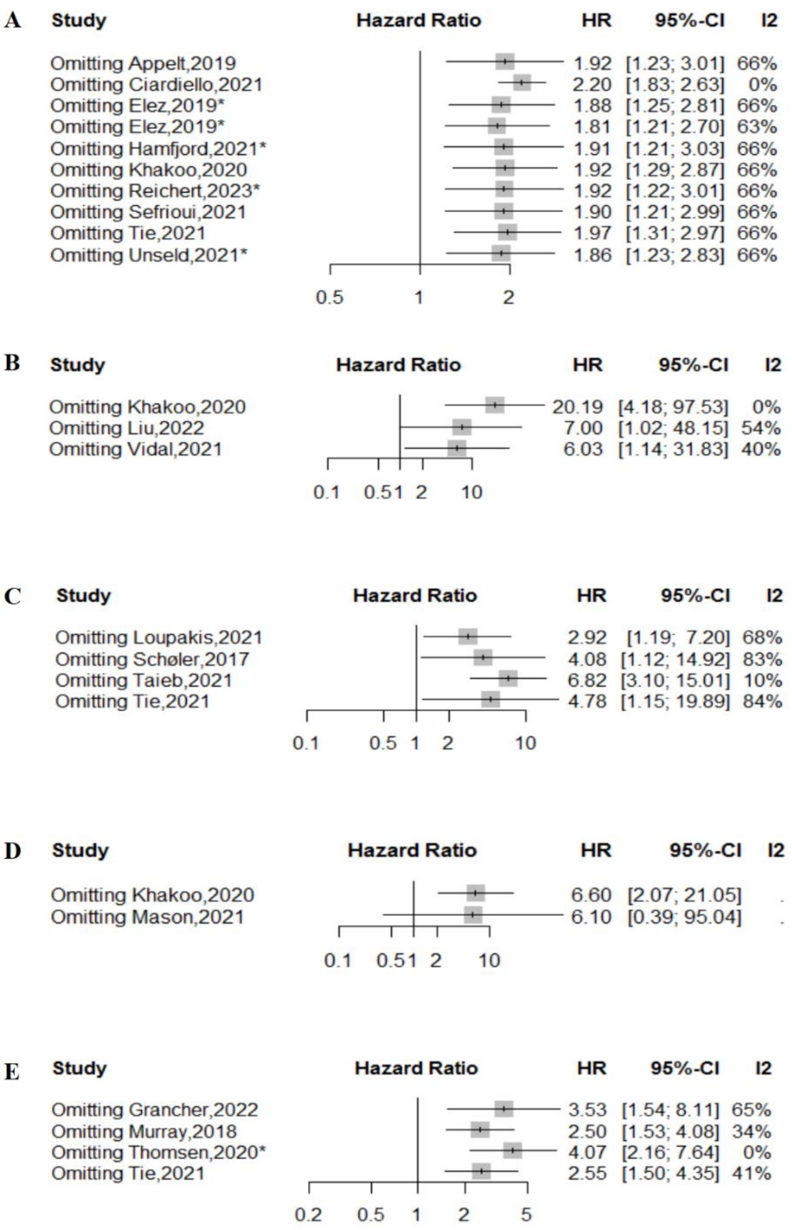

Supplementary Fig. 6 Sensitivity analysis of the association between ctDNA detection and OS using leave-one-out methods.

(A) baseline (before any treatment, all CRCs); (B) after NAT and before surgery (only for LARC); (C) after surgery (only for resectable CRCs); (D) during surgery and ACT (only for resectable and CRCs required ACT); (E) after full-course treatment (after completing corresponding treatments according to the clinical guidelines, all CRCs).

Abbreviation: ACT, adjuvant chemotherapy; CRC, colorectal cancer; CI, confidence interval; HR, hazard ratio; LARC: localized advanced rectal cancer; mCRC, metastatic colorectal cancer; NAT, neoadjuvant chemotherapy; OS, overall survival.

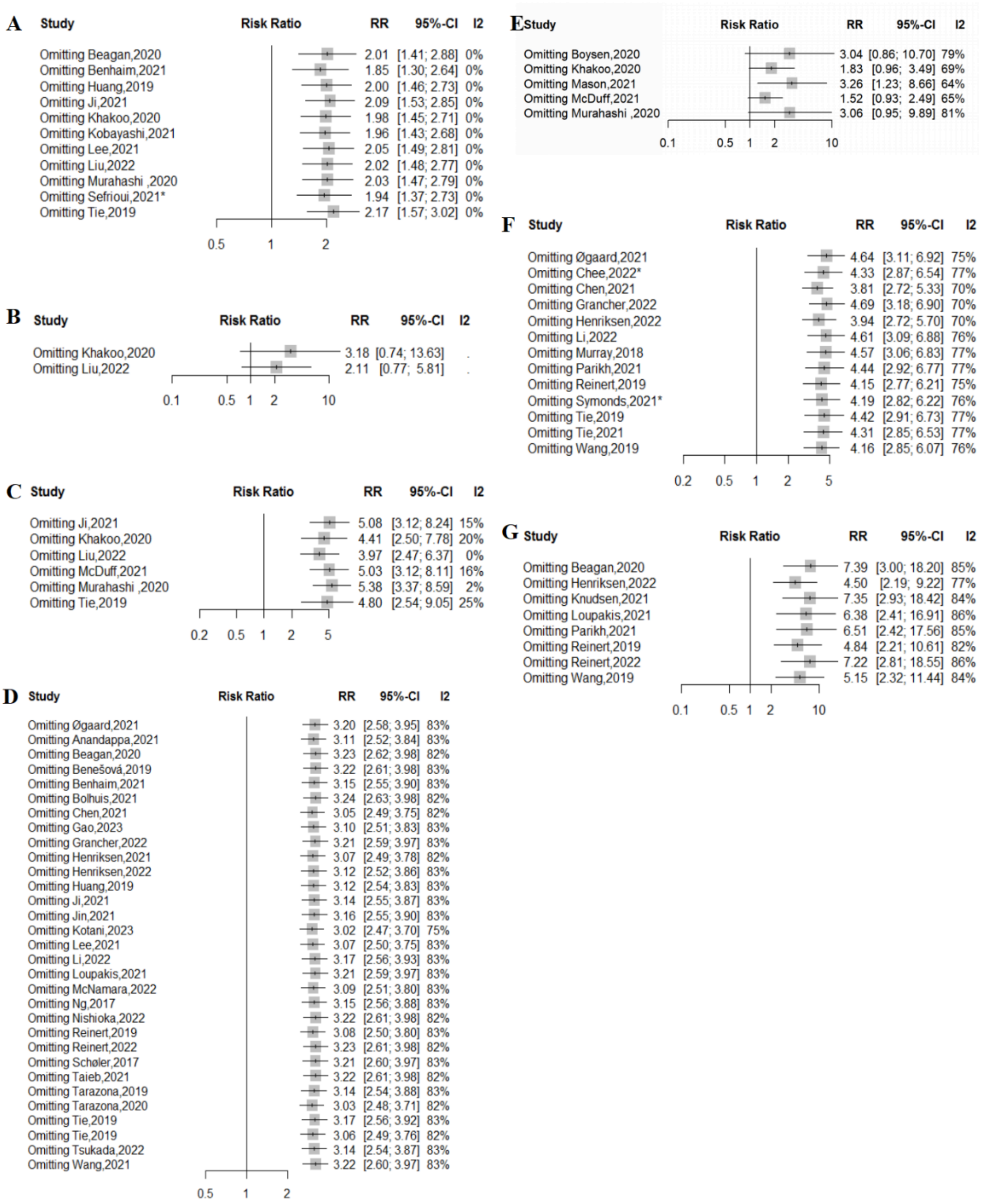

Supplementary Fig. 7 Sensitivity analysis of the association between ctDNA detection and recurrence using leave-one-out methods.

(A) baseline (before any treatment, all CRCs); (B) during NAT (only for LARC); (C) after NAT and before surgery (only for LARC); (D) after surgery (only for resectable CRCs); (E) during surgery and ACT (only for resectable and CRCs required ACT); (F) after full-course treatment (after completing corresponding treatments according to the clinical guidelines, all CRCs); (G) long-term post-treatment surveillance (all CRCs).

Abbreviation: ACT, adjuvant chemotherapy; CRC, colorectal cancer; CI, confidence interval; LARC, localized advanced rectal cancer; mCRC, metastatic colorectal cancer; NAT, neoadjuvant chemotherapy; RR, risk ratio.

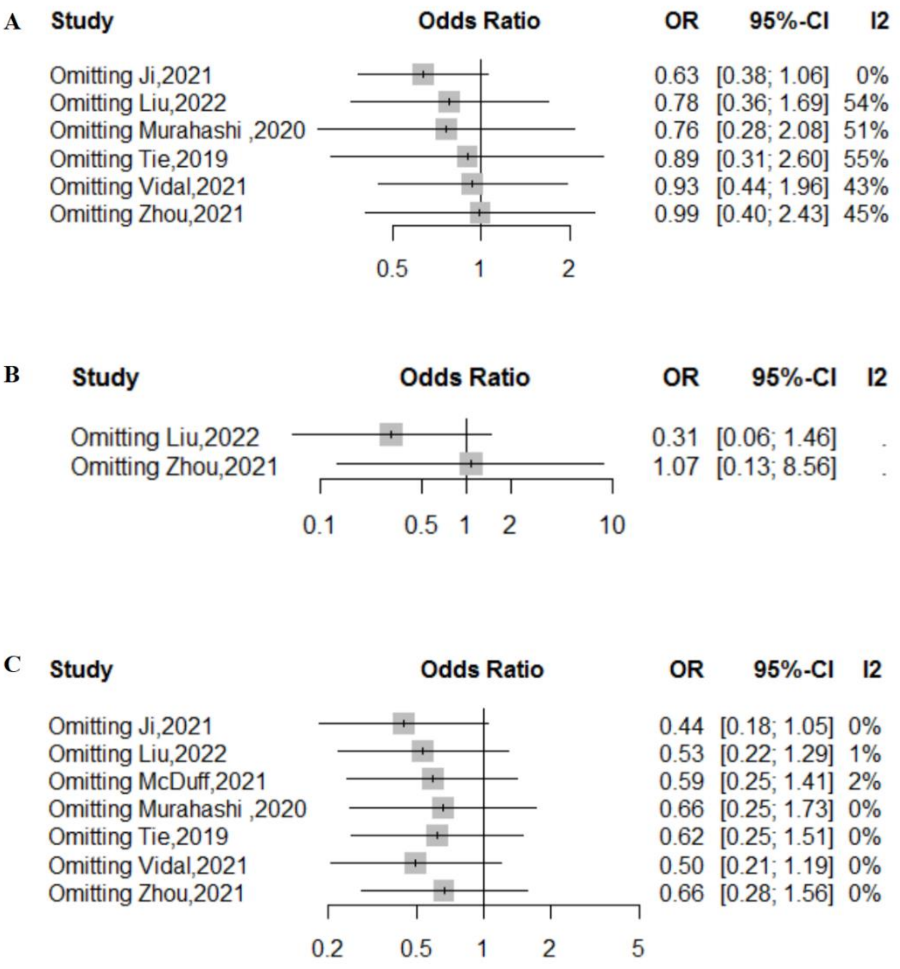

Supplementary Fig. 8 Sensitivity analysis of the association between ctDNA detection and pCR using leave-one-out methods.
(A) baseline (before any treatment, all CRCs); (B) during NAT (only for LARC); (C) after NAT and before surgery (only for LARC).

Abbreviation: CI, confidence interval; CRC, colorectal cancer; LARC, localized advanced rectal cancer; NAT, neoadjuvant chemotherapy; OR, odds ratio; pCR, pathologic complete response.

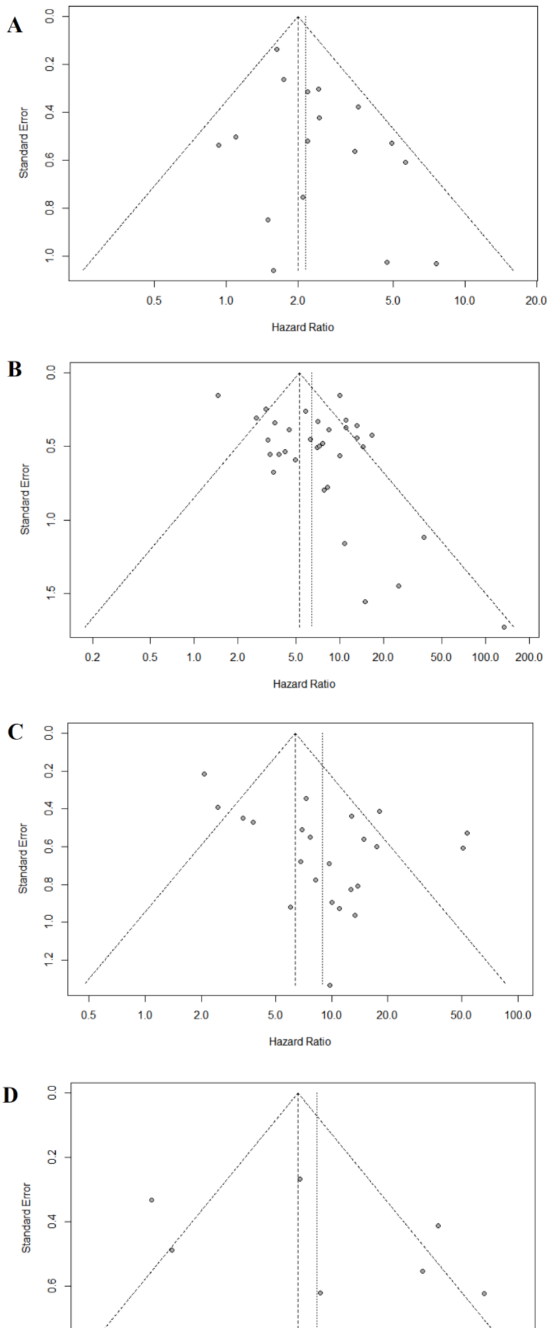

Supplementary Fig. 9 Funnel plot of the association between ctDNA detection at and RFS.

(A) baseline (before any treatment, all CRCs, $P_{\text{Egger test}}$ =0.058); (B) just after surgery (only for resectable CRCs, $P_{\text{Egger test}}$ =0.036); (C) after full-course treatment (after completing corresponding treatments according to the clinical guidelines, all CRCs, $P_{\text{Egger test}}$ =0.004); (D) long-term post-treatment surveillance (all CRCs, $P_{\text{Egger test}}$ =0.414).

Abbreviation: CRC, colorectal cancer; RFS, recurrence-free survival.

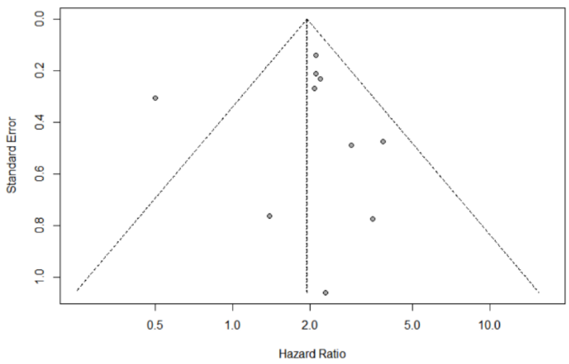

Supplementary Fig. 10 Funnel plot of the association between ctDNA detection at baseline (before any treatment, all CRCs) and OS (*P* $_{\text{Egger test}}$ =0.985).

Abbreviation: CRC, colorectal cancer; OS, overall survival.

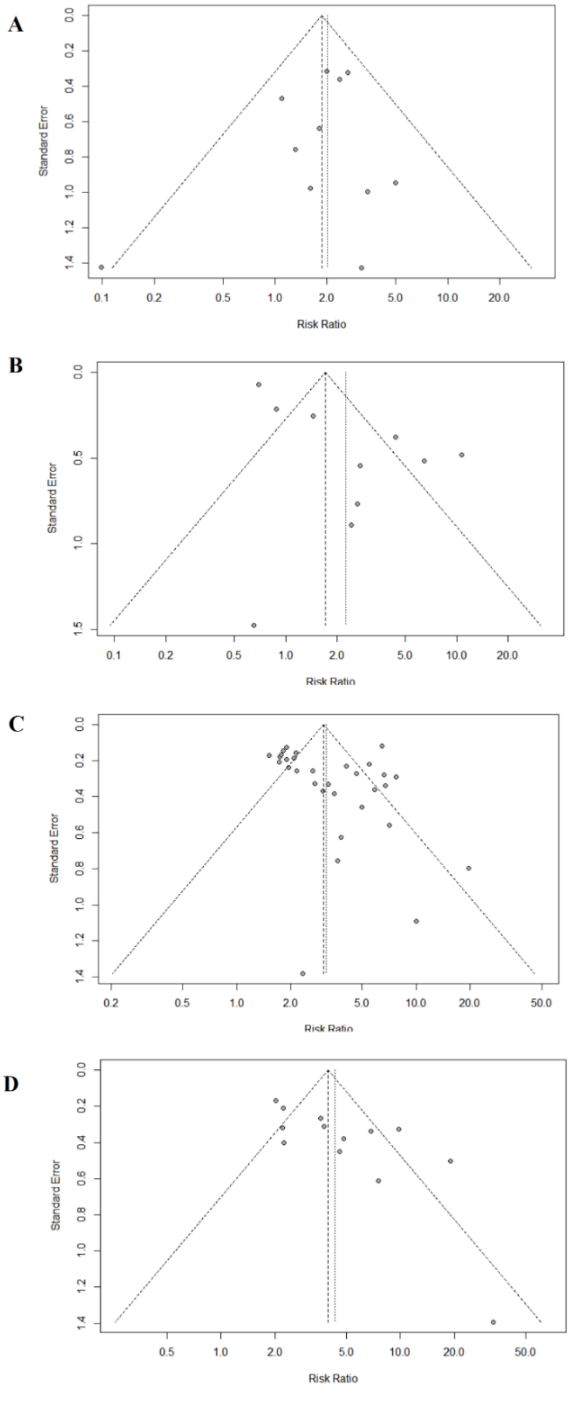

Supplementary Fig. 11 Funnel plot of the association between ctDNA detection and recurrence.

(A) at baseline (before any treatment, all CRCs, $P_{\text{Egger test}}$ =0.400); (B) after NAT and before surgery (only for LARC, $P_{\text{Egger test}}$ =0.007); (C) after surgery (only for resectable CRCs, $P_{\text{Egger test}}$ =0.085); (D) after full-course treatment (after completing corresponding treatments according to the clinical guidelines, all CRCs, $P_{\text{Egger test}}$ =0.007).

Abbreviation: CRC, colorectal cancer; LARC, localized advanced rectal cancer; NAT, neoadjuvant chemotherapy.
